# Humic substances enhance the anti-cancer efficacy of standard therapies

**DOI:** 10.1038/s41420-026-03083-1

**Published:** 2026-03-31

**Authors:** Paola Bianca, Chiara Modica, Mariavittoria Verrillo, Melania Lo Iacono, Laura Rosa Mangiapane, Kimiya Shams, Narges Roozafzay, Vincenzo Davide Pantina, Giulia Bozzari, Sebastiano Di Bella, Francesco Orilio, Caterina D’Accardo, Gaetana Porcelli, Roberta Drago, Francesco Verona, Rosario Nicola Brancaccio, Alice Turdo, Miriam Gaggianesi, Simone Di Franco, Vincenza Cozzolino, Riccardo Spaccini, Matilde Todaro, Giorgio Stassi

**Affiliations:** 1 Department of Health Promotion Sciences, Internal Medicine and Medical Specialties (PROMISE), University of Palermo, Palermo, Italy; 2 Azienda Ospedaliera Universitaria Policlinico “Paolo Giaccone” (AOUP), Palermo, Italy; 3 Department of Precision Medicine in Medical, Surgical and Critical Care (Me.Pre.C.C.), University of Palermo, Palermo, Italy; 4 Department of Agricultural Sciences, University of Naples Federico II, Naples, Italy; 5 IRCCS SDN, Naples, Italy

**Keywords:** Cancer therapy, Natural products

## Abstract

The green oncology paradigm emphasizes the use of natural products in cancer treatment to protect the environment while reducing the adverse effects associated with conventional therapies. In this context, humic substances (HSs), derived from the degradation of waste biomass, have emerged as promising candidates due to their diverse bioactive properties. Beyond their well-known antioxidant and antimicrobial effects, this study demonstrates the antitumor potential of HSs extracted from olive (HS-OL) and artichoke (HS-CYN). Our results reveal that HS-OL and HS-CYN significantly induce DNA damage by triggering apoptosis and reducing cell viability in cancer cells across various histotypes. When used in combination with standard therapies, these HSs enhance therapeutic efficacy, enabling the use of lower doses of chemotherapeutic agents while maintaining their effectiveness. The introduction of HSs into cancer treatment represents a sustainable and innovative approach that not only reduces the ecological footprint but also minimizes the side effects associated with traditional anticancer drugs, offering a dual benefit for both patients and the environment.

## Introduction

Green oncology introduces an innovative approach to cancer treatment, integrating patient-centered care with a focus on social, economic, and environmental sustainability. Unlike traditional oncology, which prioritizes individual outcomes, green oncology emphasizes eco-responsibility and the broader impact of medical practices on society and the biosphere. This paradigm seeks to balance therapeutic efficacy with ethical and sustainable practices, addressing the growing need for treatments that are not only effective but also equitable and environmentally conscious [1]. The global burden of cancer underscores the urgency of innovative approaches. In 2019 alone, approximately 23.6 million new cancer cases were diagnosed worldwide, leading to 10 million deaths and significant disability among survivors. Additionally, the economic strain is evident, with oncology drug sales reaching USD 164 billion in 2020, reflecting a rapid annual growth rate of 14.3% over five years. While conventional therapies comprising surgery, chemotherapy, and radiotherapy have improved survival rates, they are associated with substantial costs and debilitating side effects that challenge both patients and healthcare systems [2]. First-line treatment for solid tumors typically involves a combination of surgery, chemotherapy, and radiotherapy. While these approaches have improved efficacy and patient survival rates, they impose substantial costs on public health systems. Moreover, short and long-term side effects of these treatment regimens pose significant challenges for patient care and clinicians. To address these concerns, researchers have been developing new strategies that improve treatment tolerance and optimize the scheduling of radiotherapy or chemotherapy. In this context, natural products could serve as promising candidates for successful alternative therapies. Natural bioactive compounds retain unique properties related to their structure, high chemical diversity, and low toxicity, making them suitable for several biomedical applications [3]. Humic substances (HSs), derived from the decomposition of organic matter, exhibit biological activity that significantly impacts soil health, enhances plant growth, and facilitates microbial interactions [4]. The effects of HSs on human health have garnered increasing scientific attention due to their potential therapeutic advantage [5–8]. Humic materials display antioxidant, anti-inflammatory, and immune-modulating properties by regulating cytokine production, which is essential for managing inflammation and autoimmunity [9–11]. It effectively mitigates oxidative stress by scavenging free radicals, thus protecting cells from oxidative damage and potentially slowing the aging process [12]. In cancer research, HSs exhibited potential anti-cancer properties by inducing apoptosis and inhibiting the proliferation of various cancer cell lines [13]. These effects are likely attributed to their interactions with cellular signaling pathways that regulate cell growth and survival. Despite these promising findings, it is important to note that the biological activity of humic extracts may depend on their source, composition, and concentration. Therefore, rigorous standardization and quality control are essential in research and therapeutic applications.

Here, we investigated the antioxidant, antibacterial and anti-tumor potential of HSs derived from the decomposition of olive and artichoke (HS-OL and HS-CYN, respectively), on different cancer cells. The biological activity of both HS-OL and HS-CYN is primarily attributed to their high content of bioactive chemical groups, particularly polyphenols such as oleuropein and hydroxytyrosol. These compounds exhibited antioxidant, anti-inflammatory, and immunomodulatory properties [14–17].

We evaluated HS-OL and HS-CYN effectiveness as adjuvant therapies across three tumor models: colorectal, thyroid and breast cancer. Our findings demonstrate that HS-OL and HS-CYN significantly induce DNA damage, impair cell viability and enhance apoptosis in cancer cells endowed with metastatic capacity. Furthermore, their ability to sensitize tumors to standard therapies highlights their potential as sustainable adjuvants in oncology. This study reinforces the value of integrating natural products like HSs into cancer treatment protocols to improve outcomes while reducing environmental and systemic toxicity.

## Results

### Molecular characterization of humic extracts

**Fig. 1:**
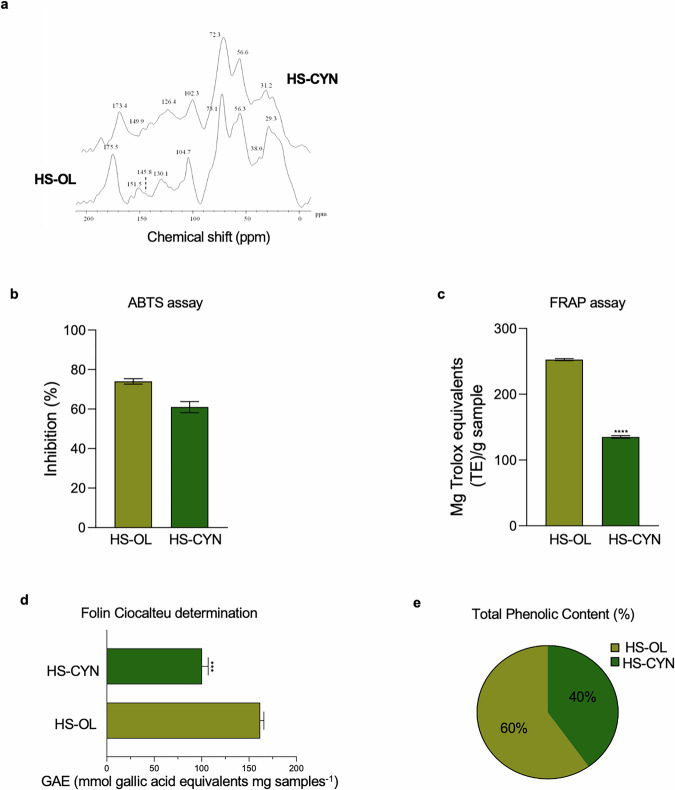
HS-OL and HS-CYN characterization. **a** 13C CPMAS NMR spectra of HS-OL and HS-CYN; **b**, **c** Antioxidant capacity of HS-OL and HS-CYN measured by ABTS (p value = 0.0095) (**b**) and FRAP (*p* value < 0.0001) (**c**), analyzed with spectrophotometric methods. Comparisons between two groups were made using the Unpaired T test. **d** Correlation between antioxidant activity of HS-OL and HS-CYN and phenolic content. **e** Representation of total phenolic composition percentage (%TPC) in HS-OL and HS-CYN.

The outputs of 13C CPMAS-NMR assessment of HSs from processed artichoke and olive pomace organic residues are shown in Fig. 1a and Table 1. The alkyl-C chemical range (0–45 ppm) revealed sharp peaks around 31/32 ppm flanked by lateral shoulders at 38 ppm, pertaining respectively to CH2 and CH bonds of linear and cyclic lipid molecules such as fatty acids, alcohols, plant biopolyesters and sterols. The prominent resonances rising at 55/56 ppm derive from the methoxyl substituents linked to the aromatic ring, indicating the effective incorporation of lignin fragments coupled with the possible contribution of C-N bonds of peptide units. The central spectrum interval (60–110 ppm) encompasses the O-alkyl-C nuclei of oligo and polysaccharides marked by the broad coalescence of pyranose ring carbons at 72 ppm and the de-shielded di-O-alkyl function of anomeric carbons around 102–104 ppm (Fig. 1a). The different bands extended in the subsequent chemical shift (110–160 ppm) are associated to overall aromatic (110–140 ppm) and O-aryl-C moieties mainly sourced from polyphenolic and lignin structures of plant tissues. The last sharp signals (160–90 ppm) gather the carbonyl groups in either basic components (e.g., amino acids, alkyl acids) or oxidized organic products. The overview of molecular features provided by the HB dimensionless structural index denoted an almost even partition of hydrophilic and hydrophobic domains in each humic extract, revealing a larger content of apolar aliphatic chains in HS-OL, with respect to the prevalence of O-alkyl compounds derived from polysaccharides in HS-CYN, shaped by the lower values of both HB and A/OA parameters (Table 1). The aromaticity index underlined the significant content of aromatic components, while the concomitant tendentially low values found for Lignin ratios suggested the preservation of phenolic components in both HS samples [10, 18].

**Table 1:** Carbon Distribution and Structural Index Analysis in ¹³C CPMAS NMR Spectra of Humic Substances.

	C=O	O-aryl-C	aryl-C	O-alkyl-C	CH_3_O/CN	alkyl-C
	(190-160)	(160-140)	(140-110)	(110-60)	(60-45)	(45-0)
**HS-CYN**	10.9	5.3	18.7	35.8	14.9	14.3
**HS-OL**	11.3	6.6	14.7	25.1	20.2	22.1
	**A/OA**	**ARM**	**HB**	**LR**		
**HS-CYN**	0.4	0.5	0.8	2.8		
**HS-OL**	0.9	0.5	1.2	3.1		

A/OA (alkyl ratio) = [(0–45)/(60–110)], ARM (aromaticity index)= (110–160)/Σ[(0–45) + (60–110)].

HB (hydrophobicity index) = [Σ(0–45) + (45–60)/2 + (110–160)]/Σ[(0–45) + (45–60)/2 + (60–110) + (160–190)].

LR (Lignin ratio)= (45–60)/(140–160)-.

Our findings establish a large range of monomers consisting of aliphatic and aromatic molecules detected as methyl esters and ethers on natural compounds of mainly plant origin and secondly of microbial by-products (Supplementary Fig. 1 and Table 2). With respect to solid-state NMR, a lower abundance of carbohydrate and polysaccharide derivatives was found in both pyrograms of humic materials. This behavior is attributed to the lower efficiency of off-line pyrolysis techniques to detect polar thermally metastable O- and N-bearing substances in complex matrices. The stability and pyrolytic rearrangement of poly-hydroxy compounds and the reaction condition of TMAH reagent solution are recognized to negatively interfere with the diagnostic identification of polysaccharides and peptidic components [19, 20].

**Table 2:** Main products identified in the THM-GCMS of humic substances.

r.t.	Compound/ Source	r.t.	Compound/ Source
7.9	1,2-diCH3O benzene/Lg G_1_	19.6	3,4,5-triCH3O benzaldehyde/Lg S_4_
9.6	3-pyridine derivative/N-Pp	20.3	Cis-2-(3,4-diCH3O phenyl)-1-CH3O- ethylene/Lg G_7_
10.1	Benzaldehyde, 4-CH3O/Lg P_4_	20.6	Trans-2-(3,4-diCH3O phenyl)-1-CH3O- ethylene/Lg G8
10.4	Methyl-indole/N-Pp	20.9	Cis-1-(3,4-diCH3O phenyl)-1-CH3O-1-propene Lg G11
10.7	2,4,5,6,7-Penta CH3O Heptanoic acid, m.e./Carb	21.3	Acido 2-propenoico,3-(4-CH3O phenyl) m.e./Lg P_18_
11.2	Carbohydrate m/z 89, 101, 129, 161/ Carb	21.6	1-(3,4,5-triCH3O phenyl)-ethyl ketone/ Lg S_5_
11.4	1,2,4-triCH3O benzene/Carb	21.9	Acido C14 m.e./Mic
10.2	N derivative m/z 98/N-Pp	22.7	Benzoic acid, 3,4,5-triCH3O m.e. /Lg S_6_
13.0	Benzene, 4-ethenyl-1,2-diCH_3_O/ Lg G_3_	22.9	Tetradecanoic acid m.e./Lip
13.1	Benzene 1,2,3-triCH_3_O/Lg S_1_	23.3	1-(3,4-diCH3O phenyl)-3-CH3O-1-propene/LgG_13_
13.3	Benzoic acid 4-CH_3_O m.e./LgP_6_	24.1	cis-1-(3,4,5-triCH3Ophenyl)-2-CH3O ethylene/Lg S_7_
13.5	1,5-anhydro-2,3,4,6-tetra-O-methyl-D-glucitol/Carb	24.3	2-propenoic acid, 3-(3,4-diCH3O phenyl) m.e./LgG_18_
13.6	1,4- anhydro -5-O-acetil-2,3,6-tri-O-metil-D-glucitol/Carb	24.6	Thr/Erith.1-(3,4-diCH3O phenyl)-1,2,3-tri CH3O propane/Lg G_14_
13.7	D-xylopiranose, -5-CH3O-2,3,4-tri-O-methyl/Carb	24.6	iso Pentanoic acid m.e./Mic
14.9	Benzene acetic acid 4-CH3O m.e/Lg P_23_	24.7	trans-1-(3,4,5-triCH3Ophenyl)-2/-CH3O ethylene/Lg S_8_
15.1	1,2,3,4-tetraCH3O benzene Carb	24.8	anteiso Pentanoic acid m.e./Mic
15.3	N heterocyclic m/z 69,97,154/N-Pp	24.9	Thr/Erith.1-(3,4-diCH3O phenyl)-1,2,3-tri CH3O propane/Lg G_15_
15.9	m/z 71, 87, 101, 129, 161 Carb	25.5	C15 n-acido m.e./Lip
16.3	Benzaldehyde, 3,4-diCH_3_O /Lg G_4_	27.0	1-(3,4-diCH3O phenyl)-1,3-diCH3O-1-propene/Lg G_16_
17.1	Isomer G7/G8/Lg G	27.1	Methyl Hexadecanoic acid, m.e./Mic
17.7	TetraCH3O benzene/Carb	27.2	Hexadecenoic acid m.e./Mic
18.0	Benzene acetic acid 3,4-diCH3O m.e./LgG_23_	27.4	Thr/Erith.1-(3,4,5-triCH3O phenyl)-1,2,3-triCH3O propane/Lg S_14_
16.6	1-(3,4-diCH3O phenyl)- ethyl -ketone/ Lg G_5_	27.7	Hexadecenoic acid m.e.
19.4	Benzoic acid, 3,4diCH3O m.e./Lg G_6_	27.8	Thr/Erith.1-(3,4,5-triCH3O phenyl)-1,2,3-triCH3O propane Lg S_15_

r.t.	Compound/ Source	r.t.	Compound/ Source
28.1	Hexadecanoic acid m.e./Lip	42.8	Tetracosanol m.et./Lip
29.0	iso Heptadecanoic acid m.e./Mic	43.3	C20 doic acid dime/Biop
29.5	anteiso Heptadecanoic acid m.e./Mic	44.1	Heptacosane/Lip
30.4	Heptadecanoic acid m.e./Lip	44.7	Tetracosanoic acid m.e./Lip
32.0	Octadecenoic acid m.e./Lip	45.2	C22 acid,22 CH3O, m.e./Biop
32.2	Octadecenoic acid m.e./Mic	46.4	Hexacosanol m.et./Lip
32.7	Octadecanoic acid m.e./Lip	47.0	C22 doic acid dim.e./Biop
33.3	C16 acid, 16CH_3_O, m.e./Biop	47.6	Nonacosane/Lip
34.5	C18 acid ciclopropane m.e./Mic	48.1	Hexacosanoic acid m.e./Lip
34.9	Eicosanol m.et./Lip	48.6	C24 acid, 24 CH3O, m.e./Biop
35.4	C16 dioic acid dim.e./Biop	49.8	Octacosanol m.et./Lip
36.2	C16 acid, 9(10)-16 diCH_3_O, m.e./Biop	50.1	Sterol (tetracyclic) m.et./Lip
36.8	C18:1 acid,18 CH_3_O, m.e./Biop	50.2	Sterol (tetracyclic) m.et./Lip
37.0	Eicosanoic acid m.e./Lip	50.7	Sterol (pentacyclic) m.et./Lip
37.5	C18 acid, 18CH_3_O, m.e. /Biop	50.8	Triacontane/Lip
38.1	C16 acid, triCH_3_O, m.e. /Biop	51.4	Octacosanoic acid m.e./Lip
38.9	C18:1 doic acid dim.e./Biop	51.8	C26 aacid, 26 CH3O, m.e./Biop ·
39.0	Docosanol m.et./Lip	52.7	Sterol (tetracyclic) m.et./Lip
39.5	C18 doic acid dim.e./Biop	52.9	Sterol (tetracyclic) m.et./Lip
41.0	Docosanoic acid m.e./Lip	55.6	Sterol (pentacyclic) m.et./Lip
41.5	C20 acid,20 CH3O, m.e./Biop	55.8	Sterol (pentacyclic) m.et./Lip

*Biop* biopolyester, *Carb* carbohydrate, *CH*_3_*O* metoxy, *dim.e*. dimethyl ester,

*Lg* lignin, *Lip* lipid, *m.e*. methyl ester, *met* methyl ether, *Mic* microbial, *r.t* retention time.

The apolar alkyl components found in HSs were represented by linear fatty acids, hydroxy-fatty acids and dioic acids, largely dominated even chain length components (C16-C28), thus suggesting the plant constituents of waxes, cutin and suberin as prevalent sources (Table 2) [4]. In line with the indication of NMR analyses, the survey of aromatic fractions highlighted the prevalence of polyphenolic derivatives inherited from the structural components that build up the lignified tissues of higher plants (Table 1). The specific monomers identified by fragmentation pattern are associated with the current symbols used to distinguish the different structural units: P, *p*-hydroxyphenyl; G, guaiacyl (3-methoxy,4-hydroxyphenyl); S, syringyl (3,5-dimethoxy, 4-hydroxyphenyl). Among lignin fragments, the identification of the enantiomers of 1-(3,4-dimethoxyphenyl)-1,2,3-trimethoxypropane (G14 and G15) and 1-(3,4,5-trimethoxyphenyl)-1,2,3-trimethoxypropane (S14 and S15), which exhibit an integral hydroxylated side chain, indicated the persistence of not decomposed lignified plant tissues. Conversely, the aldehydic (G4, S4), ketonic (G5, S5), and benzoic-acid (G6, S6) components (Table 1) result from the oxidation processes and are proxies of progressive cleavage of intermolecular bonds [10, 18]. Moreover, the concomitant release of additional phenolic structures such as 2-(3,4-di-methoxyphenyl)-1-methoxyethylene (G7/(), 1-(3,4-dimethoxyphenyl)-1(3)-methoxy-propene (G10/11, G13), 1-(3,4,5-tri-methoxy phenyl)-2-methoxyethylene (S7/8) and 1-(3,4,5-trimethoxyphenyl)-1(3)-methoxypropene (S10/11, S13), as either cis or trans isomers may be related to the incorporation of intermediate derivatives of depolymerization processes. In this respect, besides the relative abundance of lignin components, which is partially undermined by the lack of carbohydrate, these biomarkers produced by thermochemolysis allow the evaluation of structural parameters used to estimate the extent of lignin decomposition and potential bioavailability. Therefore, the ratio of acidic structures over that of, both, the corresponding aldehydes (Ad/AlG=G6/G4, Ad/AlS=S6/S4) and over the sum of peak areas for the threo/erythro isomers (ΓG = G6/ [G14 + G15]; ΓS = S6/[S14 + S15]), are considered to be reliable indicators of the oxidative transformation of lignin polymers.

The data of decaying index displayed by lignin compounds (Table 3) highlighted the occurrence of prevalent lignin depolymerization with less oxidative degradation during the composting activities, which promoted the subsequent incorporation of a wide array of partially decomposed bioavailable phenolic fragments in HS-CYN and HS-OL.

**Table 3:** Compound classes (%) of THM and lignin decaying index^a^.

	HS-CYN	HS-OL
Carbohydrates+ N compound	15.7	10.9
Lignin	65.1	49.6
Lipid	19.7	39.5
Ad/Al_G_	2.8	1.9
Ad/Al_S_	2.9	2.0
Γ_G_	2.2	2.7
Γ_S_	2.4	2.6

Ad/Al = G6/G4, S6/S4; Γ=G6/(G14 + G15), S6/(S14 + S15).

### HSs display antioxidant and antibacterial activity

To gain a more comprehensive characterization of HSs and to support the valorization of agro-industrial residues as sustainable sources of functional biomaterials, we assessed the antioxidant and antibacterial activities of the extracted HSs. ABTS assay revealed higher percentage inhibition values for compost extracts from olive compared to those from artichoke (Fig. 1b). Specifically, HS-OL showed 73% inhibition, while HS-CYN exhibited 64%. When expressing the ABTS antioxidants as TEAC (mmol Trolox equivalent per kg of the sample), HS-OL had a value of 408.5, followed by HS-CYN with 380. This comparison highlights the antioxidant capacity of the humic extracts against a standard antioxidant compound. A similar trend was noted by the FRAP assay, measuring the total antioxidant capacity as mg Trolox equivalents per gram of samples (Fig. 1c). This spectrophotometric determination confirmed that HS-OL had a greater antioxidant capacity than HS-CYN, with values of 254 and 127.5, respectively. In line with previous studies, the antioxidant activity of HS-OL and HS-CYN correlated directly with their total phenolic content, as measured by the Folin-Ciocalteu assay (Fig. 1d). The phenolic composition was also significant, with percentages of 60% and 40% for HS-OL and HS-CYN, respectively, as shown in Fig. 1e. Although the Folin-Ciocalteu reagent is not specific for polyphenols, our findings align with previous research suggesting a strong correlation between total phenolic content and antioxidant capacity in green compost extracts. Moreover, compounds exhibiting greater antioxidant activity were consistent across samples and showed high total phenol content, with values of 156 and 103 GAE (mmol gallic acid equivalents per mg sample) for HS-OL and HS-CYN, respectively (Fig. 1d).

Among the various therapeutic properties attributed to natural products, driven by a high content of bioactive metabolites, their antimicrobial activity has been extensively studied [MEP_L_*bib*^13^, ^13^]. Here, we evaluated the antimicrobial efficacy of olive and artichoke HS by the diffusion disk (DDK) method. In our screening, HSs derived from green compost were tested against certain Gram-positive (*S. epidermis* and *L. monocitogenes*) and Gram-negative bacterial strains (*H. pylori* and *S. typhi*), while a protein such as bovine serum albumin (BSA) or a common antibiotic such as ampicillin was used as a negative or positive reference control. Based on the analysis of the extracts tested, a strong antibacterial activity was observed against Gram-positive bacterial strains defined as multi-drug resistant, such as *S. epidermis*, with an inhibition of 9.4 mm for HS from olive trees, while 6.3 in the case of HS from artichoke (Table 4). Our findings establish olive HS as a novel, sustainable source of multifunctional bioactive materials, with previously unreported antimicrobial properties against *Helicobacter pylori* and *Salmonella typhi* implicated in the development of several inflammatory diseases. Clear antimicrobial activity has been exhibited from natural compounds (e.g., essential oil derived from Aquifoliaceae, Malpighiaceae) against *H. pylori* and *S. typhi*, two different microbial strains involved in the pro-tumoral inflammation microenvironment in gastric cancer [21, 22]. This contribution expands the range of agro-industrial residues that can be valorized for biological applications. By comparing HS from olive and artichoke feedstocks, we further demonstrate that their bioactivity is highly dependent on the biomass origin and composting conditions and the abundance of aromatic and phenolic fractions in these products. Notably, the observed antimicrobial activity, particularly in light of growing evidence connecting microbiota dysbiosis to cancer progression, may add to the therapeutic relevance of HS in oncological contexts.

**Table 4:** Antimicrobial analysis of HS-OL and HS-CYN.

Diameter(mm)	S.epidermis	L.monocitogenes	H.pylori	S.thyphi
**HS-CYN**	6.3 ± 0.03b	7.1 ± 0.06b	7.5 ± 0.01b	7.9 ± 0.04b
**HS-OL**	9.4 ± 0.05c	8.3 ± 0.02c	8.1 ± 0.05c	8.5 ± 0.08c
**BSA**	n.i.	n.i.	n.i.	n.i.
**AMP+ Clavulanic acid**	5.1 ± 0.01a	4.2 ± 0.04a	3.5 ± 0.01a	4.8 ± 0.02a

### Exposure to HS-OL and HS-CYN affects cancer cell survival

**Fig. 2:**
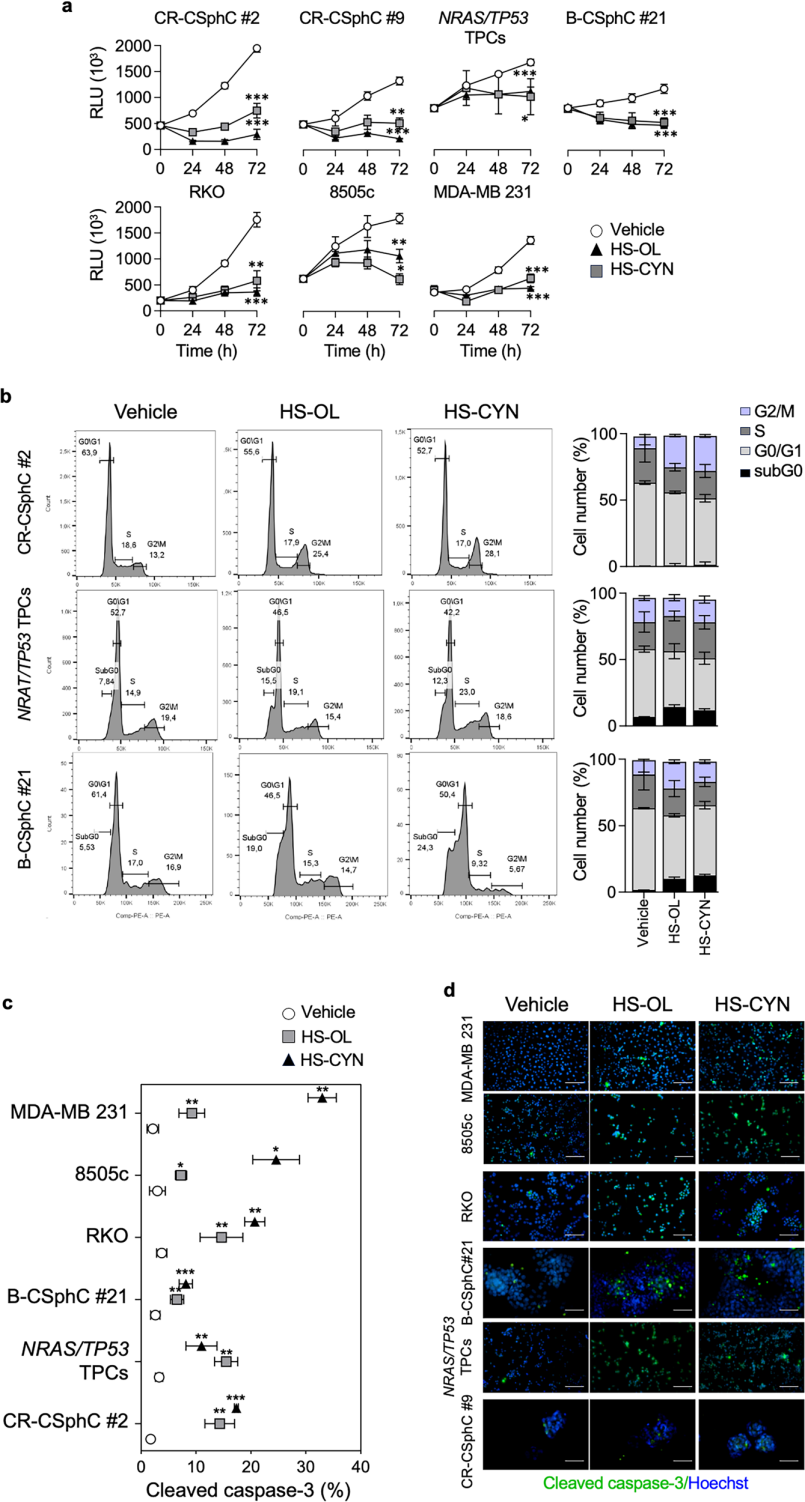
HS-OL and HS-CYN treatment hampers cancer proliferation and induces apoptosis. **a** Proliferation assay, assessed by Cell Titer Glo, in colorectal (CR-CSphC #2, CR-CSphC #9), thyroid (*NRAS/TP53* TPCs), breast (B-CSphC #21) cancer cells (*Upper panel*) and colorectal (RKO), thyroid (8505c) and breast (MDA-MB 231) established cancer cell lines (*Bottom panel*), treated with vehicle, HS-OL or HS-CYN, up to 72 h. CR-CSphC #2, CR-CSphC #9, RKO and 8505c cell lines were treated with 500 ug/ml of HS-OL or HS-CYN; *NRAS/TP53* TPCs cells were treated with 125 ug/ml of HS-OL or HS-CYN; B-CSphC #21 and MDA-MB 231 cell lines were treated with 350 ug/ml of HS-OL or HS-CYN. Data are represented as mean ± SD of three different experiments. **b** Cell cycle analysis in CR-CSphC #2, *NRAS/TP53* TPCs and B-CSphC #21 primary cell lines treated with vehicle, HS-OL, HS-CYN for 48 h as in (**a**) (*Left panel*). The percentages of cells in sub‑G0, G1, S, and G2 phases are shown as mean ± SD from two independent experiments (*Right panel*). **c** Activated-3 Caspase flow cytometry analysis, in colorectal (CR-CSphC#2, RKO), thyroid (*NRAS/TP53* TPCs, 8505c), and breast cancer cells (B-CSphC #21, MDA-MB-231), treated for 48 h as in (**a**). Data are represented as mean ± SD of three different experiments. **d** Immunofluorescence analysis in colorectal (CR-CSphC #9, RKO), thyroid (*NRAS/TP53* TPCs, 8505c), and breast cancer cells (B-CSphC #21, MDA-MB-231), treated for 48 h as in (**a**). Nuclei were counterstained by Hoechst. Scale bars, 100 µm. The images are representative of three independent experiments. Comparisons between two groups were made using a two-tailed Student’s *t* test: ns, not significant; * *p* ≤ 0.05; ** *p* ≤ 0.01; *** *p* ≤ 0.001, **** *p* ≤ 0,0001.

We sought to evaluate the anti-tumor effects of HS-OL and HS-CYN in three distinct models, specifically colorectal, thyroid and breast cancer. We investigated the sensitivity of primary cancer sphere cells, including two of colorectal (CR-CSphC #2 and CR-CSphC #9) and one of breast (B-CSphC #21), to the treatment with HS-OL and HS-CYN. The CSphC lines mentioned are part of our extensive collection that faithfully represent the genomic and transcriptomic profiles of the original cancer cells. CSphCs represent a critical subpopulation within tumors, significantly contributing to tumor initiation, metastasis and resistance to treatment [23–26]. The study was also extended to double-mutated thyroid progenitor cells (*NRAS/TP53* TPCs), which recapitulate in vitro and in vivo the features of the anaplastic thyroid carcinoma (ATC). These cells were generated by differentiating human embryonic stem cells (hESCs) into TPCs and then using CRISPR-Cas9 technology to introduce the indicated mutations [27, 28]. Additionally, we examined 3 highly metastatic established cancer cell lines, the RKO, 8505c and the MDA-MB-231 derived from colorectal cancer (CRC), ATC and breast cancer (BC), respectively. HS-OL and HS-CYN showed a great antiproliferative effect on both primary and established cell lines, in a time- and concentration-dependent manner (Fig. 2a and Supplementary Fig. 2a). As healthy control HUVEC, IMEC, CRL1790 and WA09 cells were exposed to HS-OL and HS-CYN, exhibiting only slight toxic effects (Supplementary Fig. 2b). Thus, the ability of HS-OL and HS-CYN treatment to significantly reduce the viability of cancer cells endowed with metastatic properties is paralleled by a minimal effect on healthy cells.

### HS-OL and HS-CYN induce DNA damage

**Fig. 3:**
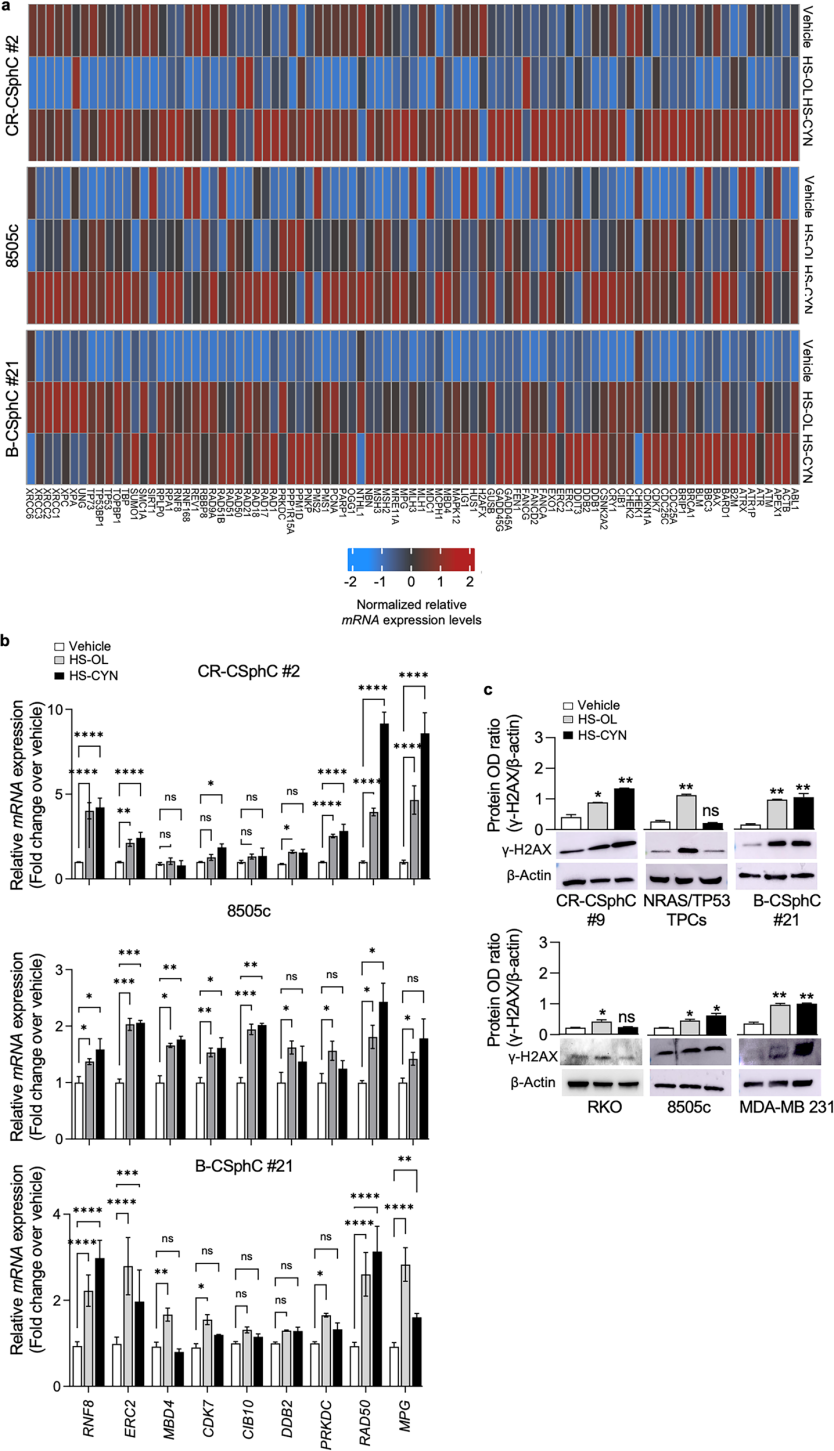
HS-OL and HS-CYN activate DNA damage machinery in colon, thyroid and breast cancer cells. **a** Heatmap of DNA damage-related genes (2^−ΔΔCt^ expression values) in colorectal (CR-CSphC #2), thyroid (8505c) and breast cancer cells (B-CSphC #21), exposed for 48 h to HS-OL and HS-CYN (*Upper panels*). GO analysis of over-expressed common genes between the treatment with HS-OL and HS-CYN in colorectal, thyroid and breast cancer cells (*Lower panels*). CR-CSphC #2 and 8505c cells were treated with 500 ug/ml of HS-OL or HS-CYN; B-CSphC #21 cells were treated with 350 ug/ml of HS-OL or HS-CYN. **b** Relative fold change over vehicle of *RNF8, ERC2, MBD4, CDK7, CIB10, DDB2, PRKDC, RAD50* and *MPG* in colorectal (CR-CSphC #2), thyroid (8505c), and breast cancer cells (B-CSphC #21), treated as in (**a**). Data are represented as mean ± SD of three different experiments. **c** Blot analysis (*upper panel*) and OD ratio (*lower panel*) of γ-H2AX in colorectal (CR-CSphC#9, RKO), thyroid (NRAS/TP53 TPCs, 8505c), and breast cancer cells (B-CSphC #21, MDA-MB-231), treated as in (**a**). Data are represented as mean ± SD of three different experiments. Comparisons between two groups were made using a two-tailed Student’s *t* test: ns, not significant; * *p* ≤ 0.05; ** *p* ≤ 0.01; *** *p* ≤ 0.001, **** *p* ≤ 0,0001.

To investigate whether HSs might be related to changes in cell cycle progression and the induction of apoptosis, we analyzed the cell cycle by flow cytometry following exposure of colon, thyroid and breast cancer cell lines to HS-OL and HS-CYN. Following treatment with HSs, colon cancer cells showed increased events in the G2/M phase arrest, according to the behavior of several anticancer agents, including alkaloids, chemotherapeutics, and antibiotics, such as nortopsentin, temozolomide and geldanamycin, which are known to induce G2/M arrest leading to cell death [29–35]. Instead, in thyroid and breast cancer cells, we observed an increase in the sub-G0 phase, caused by the HSs treatment, hallmarks of programmed cell death mediated by DNA fragmentation [36–39] (Fig. 2b and Supplementary Fig. 2c). Here, we demonstrated that treatment with HS-OL and HS-CYN led to an increase in Caspase-3 levels, indicating activation of the apoptotic pathway in all the cancer cell models analyzed (Fig. 2c, d, e and Supplementary Fig. 2d). Compelling evidence show that genotoxic stress prevents the replication of damaged DNA by activating checkpoint pathways that temporarily pause the cell cycle, allowing time for repair or, if necessary, initiating apoptosis [40]. Therefore, we evaluated the DNA repair capacity of CR-CSphC #2, 8505c and B-CSphC #21 following treatment with HS-OL and HS-CYN. A significant upregulation of DNA repair-related genes was observed after 48 h of treatment (Fig. 3a). In line with these data, the transcriptomic analysis highlighted 14 upregulated common genes between CSphCs and 53 upregulated common genes between established cancer cell lines, following treatment with HS-OL and HS-CYN, respectively. Among them, 9 genes involved in DNA repair, DNA damage, cell cycle and cell division function are commonly upregulated in response to both HS treatments (Fig. 3b and Supplementary Fig. 3a).

Notably, RAD50 upregulation is likely associated with the DNA damage checkpoint and the double-strand break repair system [41, 42]. It is well-established that various biological agents, as well as chemical and physical factors, can induce DNA double-strand breaks. These breaks activate DNA damage response (DDR), which is characterized by the phosphorylation of the histone variant H2AX, triggering cell cycle arrest and the activation of checkpoint proteins. Treatment with HSs significantly increased H2AX phosphorylation (Fig. 3c and Supplementary Figure 3b), indicating DNA damage and activation of repair machinery. These findings suggest that DNA damage, caused by HSs, overcomes the repair mechanisms, ultimately leading to the activation of apoptotic pathways.

### HS-OL and HS-CYN sensitize cancer cells to conventional therapies

**Fig. 4:**
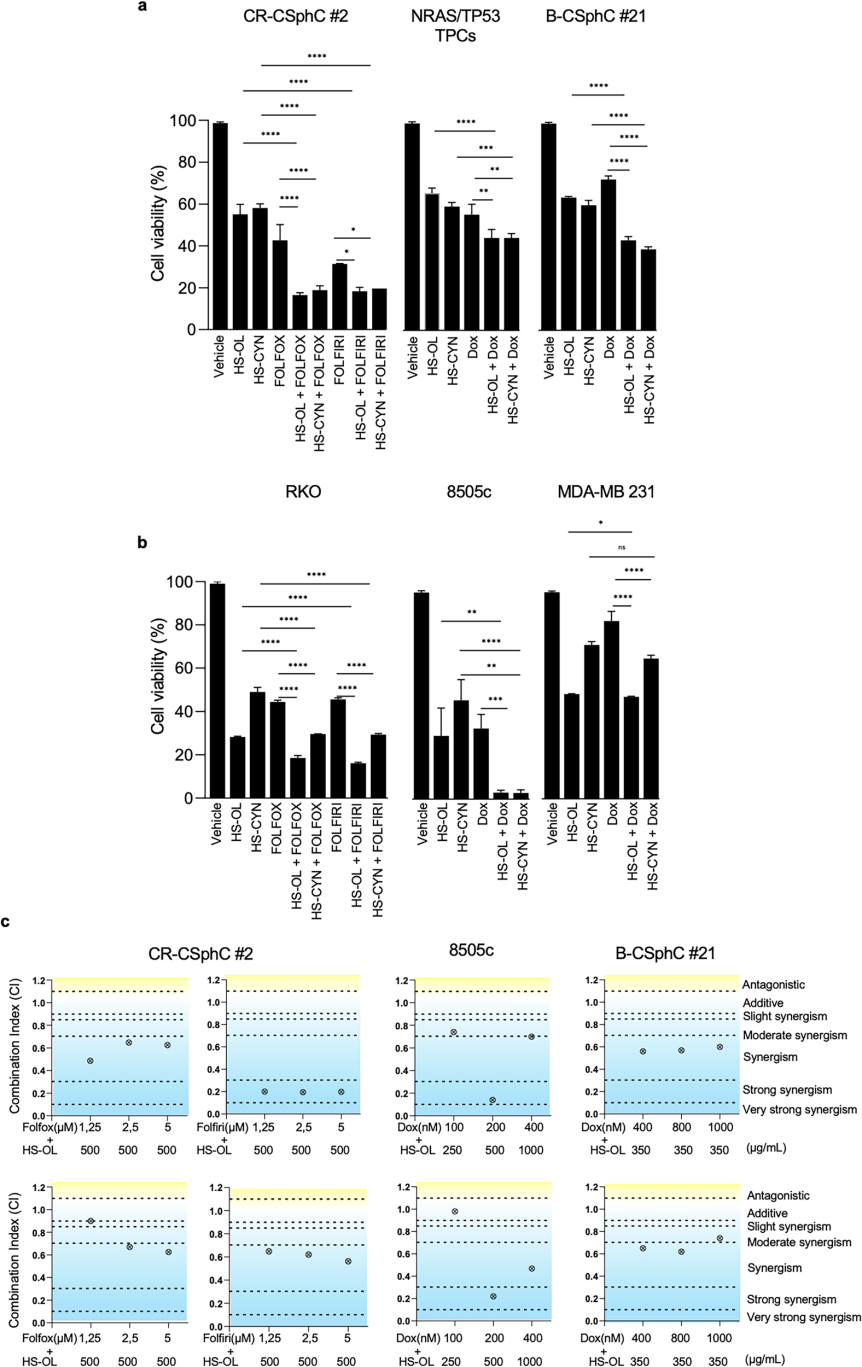
HS-OL and HS-CYN enhance the anti-tumor effect of chemotherapy or radiotherapy in colon, thyroid and breast cancer cells. **a**, **b** Cell viability analysis of colorectal (CR-CSphC #2), thyroid (*NRAS/TP53* TPCs) and breast (B-CSphC #21) cancer cells (*Upper panel*) and colorectal (RKO), thyroid (8505c) and breast (MDA-MB 231) established cancer cell lines (*Bottom panel*), treated for 48 h. CR-CSphC #2 and RKO cell lines were treated with 500 µg/ml of HS-OL or HS-CYN alone or in combination with 1,25 μM of 5-Fluorouracil, 1,25 μM of Oxaliplatin and 1,25 μM of Leucovorin (FOLFOX) or 1,25 μM of 5-Fluorouracil, 1,25 μM of Irinotecan and 1,25 μM of Leucovorin (FOLFIRI); *NRAS/TP53* TPCs were treated with 125 µg/ml of HS-OL or HS-CYN alone or in combination with 50 µM of doxorubicin; B-CSphC #21 cells were treated with 350 µg/ml of HS-OL or HS-CYN alone or in combination with 50 µM of doxorubicin; 8505c cells were treated with 500 µg/ml of HS-OL or HS-CYN alone or in combination with 200 µM of doxorubicin; MDA-MB231 cells were treated with 350 µg/ml of HS-OL or HS-CYN alone or in combination with 200 µM of doxorubicin. Data are represented as mean ± SD of three independent experiments. Comparisons between two groups were made using a two-tailed Student’s *t* test: ns, not significant; * *p* ≤ 0.05; ** *p* ≤ 0.01; *** *p* ≤ 0.001, **** *p* ≤ 0,0001. **c** Synergic plots representing the combination index (CI), according to the Chou-Talalay method, calculated by CompuSyn software, of CR-CSphC #2, 8505c and B-CSphC #21 treated as in (**a**, **b**). Data are represented as mean ± SD of two independent experiments.

While chemotherapy and radiotherapy remain standard cancer treatments, cancer cells frequently develop resistance, leading to tumor recurrence and treatment failure. Side effects associated with chemotherapy can significantly reduce patients’ quality of life, underlining the urgent need for more effective and better-tolerated therapeutic strategies. Our study demonstrated that combining either HS-OL or HS-CYN with reduced doses of FOLFOX and FOLFIRI in colon cancer cells achieved anti-tumor effects superior to those observed with higher doses of standard therapy alone, in both CR-CSC #2 and RKO cancer cells (Fig. 4a, b and Supplementary Fig. 4a). This combination strategy offers the potential to maintain therapeutic efficacy while minimizing the adverse effects typically associated with standard-dose chemotherapy regimens.

We observed similar enhanced anti-tumor effects when doxorubicin was administered in combination with HSs in both thyroid and breast cancer cells, CSphCs and established cell lines (Fig. 4a, b and Supplementary Fig. 4a). The combination of either HS-OL or HS-CYN with standard treatments demonstrated synergistic effects across all tumor models tested (Fig. 4c). Although the therapeutic combination elicits a synergistic effect in tumor cell lines treated with chemotherapy plus HSs, it exerts a negligible impact on the viability of control cells (CRL1790, IMEC, and WA09) (Supplementary Fig. 5a, b).

Our experiments revealed that combining HS-OL or HS-CYN with radiation exposure (5 Gy) significantly reduced the viability of an established cell line derived from ATC (Supplementary Fig. 4c). This synergy was further evidenced by increased caspase-3 activation in cancer cells treated with combination therapy (Supplementary Fig. 4b–d). These results strongly suggest that HS-OL and HS-CYN could serve as effective adjuvant compounds alongside conventional radio-chemotherapy in the treatment of advanced colorectal, thyroid, and breast cancers, offering a sustainable therapeutic approach.

## Discussion

Despite significant advances in therapeutic strategies, metastatic disease remains the primary cause of cancer-related deaths, largely due to its complexity and resistance to current treatments. The development of effective treatments for metastasis presents unique challenges, as metastatic cells often develop resistance to therapy [42–44]. Natural compounds offer promising therapeutic potential due to their accessibility, efficacy, and minimal side effects, largely attributed to their rich content of bioactive metabolites [45–47].

HSs in general are easily extractable from natural sources and could offer cost-saving and environmentally friendly therapeutic options. The diverse composition of HSs provides structural features that influence their bioavailability, bioactive properties, and pharmaceutical applications [47–50]. The colloidal nature and the complementary inclusion of hydrophilic and hydrophobic domains are regarded as a peculiar characteristic for the bioactive properties of humic materials. The bioactivity of HSs is determined by effective incorporation of bioavailable specific molecules (e.g., polyphenols) combined with the compositional factors -including the balance of hydrophilic and hydrophobic domains, supramolecular organization, and conformational flexibility- which play a crucial role in modulating their bioactive interactions. The pliable conformation of humic micelle-like aggregates may foster the adhesion to membranes, cells, and compartments, followed by a dynamic rearrangement and unfolding of humic aggregates. These molecular patterns are supposed to trigger the cellular signaling strengthened by the conveyance and release towards cell receptors, of carried bioactive compounds [45]. Among the various therapeutic properties attributed to natural products, antimicrobial activity has been extensively studied and remains a key focus in the search for alternatives to conventional antibiotics. In this context, we evaluated the antimicrobial potential of HSs derived from green composted olive and artichoke residues, two agro-industrial byproducts with high relevance for sustainable biovalorization. Our research demonstrates that HSs possess significant therapeutic properties, with HS-OL showing superior antioxidant capacity compared to HS-CYN through higher ABTS inhibition and FRAP values. The antioxidant properties described could be particularly relevant due to the central role of oxidative stress in inflammation, carcinogenesis, and other pathophysiological processes, suggesting that HSs may exert protective effects in these contexts. In parallel, the assessment of antibacterial activity responds to the growing need for alternative antimicrobial agents, especially those derived from sustainable sources. Moreover, given emerging links between microbial dysbiosis and cancer progression, particularly in colorectal and breast cancers, the antimicrobial potential of HSs may have indirect implications for oncological applications. For instance, in colorectal cancer, the enrichment of pathogenic species such as *Fusobacterium nucleatum*, *enterotoxigenic Bacteroides fragilis*, and *colibactin-producing Escherichia coli* has been linked to tumorigenesis, inflammation, and therapy resistance [51]. Similarly, emerging evidence in breast and thyroid cancers highlights the influence of microbiota composition on estrogen metabolism, immune responses, and clinical outcomes [52, 53]. Our findings demonstrate that both HS types exhibit antibacterial activity; however, HS-OL shows significantly greater efficacy, particularly against Staphylococcus epidermidis. This Gram-positive bacterium is known for its multidrug resistance and its ability to form biofilms within mammary gland tissues, where it disrupts host immune responses and promotes tumor cell proliferation through inflammation [54]. In addition to their action against Gram-positive bacteria, HS-OL also demonstrated inhibitory effects against *Helicobacter pylori* and *Salmonella typhi*, two Gram-negative pathogens implicated in gastric cancer development through their involvement in chronic inflammation and microbial dysbiosis. Furthermore, accumulating evidence indicates that Staphylococcus epidermidis can reshape the mammary tumor–associated microbiota toward an immunosuppressive microenvironment, whereas microbiota-targeted interventions restore antitumor immune responses [55]. In parallel, alterations in the gut microbiota, including the presence of Helicobacter pylori, have been linked to colorectal cancer risk and may influence breast cancer progression through mechanisms involving DNA damage repair or the activation of protumorigenic signaling pathways [56, 57]. This observation highlights the potential role of HS-OL, which is particularly relevant given the increasing recognition of the microbiota’s role in modulating cancer initiation and progression. Further mechanistic studies will be essential to elucidate the specific molecular features responsible for the observed activities and to explore the therapeutic potential of HSs in microbiota-associated disease contexts, including cancer. Finally, we extended the analysis of HSs on cancer cells, revealing a complex interplay of molecular mechanisms and cellular responses. Our research demonstrates that HSs exhibit significant cytotoxic effects against various cancer cell types while showing minimal toxicity to normal cells. This selective toxicity appears to be mediated through multiple cellular pathways, including ROS generation, DNA damage induction, and activation of apoptotic cascades.

The anti-cancer properties of HSs operate through several interconnected mechanisms. The compounds trigger DNA damage responses, leading to cell cycle arrest and subsequent apoptosis in cancer cells. This is evidenced by increased H2AX phosphorylation and elevated expression of DNA repair genes. HSs are more likely to exert their effects primarily through modulation of ROS, as oxidative stress is a well-established upstream driver of DNA damage and apoptotic signaling.

Oxidative stress is now widely recognized as an important predisposing factor in malignant transformation. A tightly controlled redox balance is essential not only for normal cellular homeostasis but also for the maintenance of oncogenic signaling pathways, including MAPK/ERK, PI3K/AKT, and NF-κB, which depend on physiological ROS levels to sustain pro-survival and proliferative signals [58, 59]. Importantly, both excessively high and abnormally low ROS levels can compromise cancer cell fitness, underscoring the dual role of ROS as drivers of tumorigenesis and as exploitable vulnerabilities for therapeutic intervention.

Accumulating evidence further indicates that numerous naturally occurring bioactive compounds, particularly those enriched in phenolic moieties, exert significant antitumor effects through modulation of cellular redox homeostasis [60]. Phenolic compounds possess strong antioxidant properties, enabling them to scavenge free radicals and to modulate the activity or expression of endogenous antioxidant enzymes. Through these mechanisms, they influence intracellular redox balance, attenuate oxidative damage, and interfere with signaling networks that promote malignant transformation and cancer cell survival [61].

Nevertheless, it is important to acknowledge that several studies have also demonstrated that naturally derived compounds and plant extracts can induce direct DNA damage. These effects include the induction of single- and double-strand breaks, oxidative base modifications, and DNA fragmentation, often resulting from the intrinsic chemical reactivity of specific bioactive constituents. In some cases, these compounds interact directly with DNA through intercalation or covalent binding, while in others they interfere with DNA topology or replication, ultimately compromising genomic integrity. Such direct genotoxic effects have been reported for a variety of natural products, underscoring the importance of carefully evaluating their DNA-damaging potential alongside their therapeutic or biological activities [62, 63]. Taken together, the available evidence indicates that both mechanisms, ROS modulation and direct DNA damage, may occur in response to HS exposure. The relative contribution of each pathway is likely to depend strongly on the specific chemical composition of the extracts, including the nature, concentration, and reactivity of their bioactive constituents.

Our findings indicate that HSs, specifically HS-OL and HS-CYN, can synergistically enhance the antitumor efficacy of chemotherapy administered at conventional doses. This interaction offers a dual advantage: it allows for the reduction of chemotherapeutic dosages while maintaining therapeutic efficacy, or alternatively, it amplifies the antitumor effects at standard dosing regimens. Such properties are particularly advantageous, as HSs facilitate the achievement of maximal antitumor activity at lower drug concentrations, thereby mitigating treatment-associated toxicity. Notably, the synergistic effect between HSs and conventional therapies is especially pronounced when combined with radiation therapy, wherein HSs increase the radiosensitivity of cancer cells, enhancing radiation-induced cytotoxicity and ultimately improving therapeutic outcomes. Of note, HSs exhibit selective cytotoxic effects on cancer cells while exerting minimal impact on normal cells. This selectivity may be partly explained by enhanced uptake of HSs in malignant cells, which frequently display increased endocytic activity and altered plasma membrane composition compared with their normal counterparts [64, 65]. Furthermore, differences in basal proliferation rate and DNA repair capacity may also contribute, since rapidly dividing tumor cells are more susceptible to DNA damage and replication stress than quiescent or slowly proliferating normal cells [66].

The ability of HSs to potentiate established cancer treatments carries significant clinical implications. By permitting dose reductions of chemotherapeutic agents without compromising efficacy, this approach has the potential to lessen the incidence and severity of adverse effects, thereby improving patients’ quality of life during treatment. This strategy is particularly relevant in the adjuvant setting for advanced malignancies, where optimizing therapeutic efficacy while minimizing toxicity remains a paramount clinical objective.

## Materials and methods

### Sources and processing of humic substances

Artichoke and olive agricultural wastes were collected from local composting facilities and processed to obtain green compost. Humic substances (HS-OL and HS-CYN) were then extracted following the procedure described by Verrillo et al. (2025) [67]. Conversely, compost from olive tree residues (pomace and leaves) was produced by the CNR-ISAFOM in Perugia through a prototype composting plant. The prototype comprises two 30 L bioreactors capable of controlling a “closed vessel” system for composting, although it takes place as an “industrial” type process with a typical sequence of mesophilic-thermophilic phases. Humic substances (HS-OL and HS-CYN) were then extracted following the procedure described by Verrillo et al. [13, 67]. All HS products were freeze-dried before analysis.

### ^13^C NMR spectroscopy

Solid-state ^13^C Cross-Polarization Magic-Angle-Spinning (CPMAS) NMR spectra of HSs were recorded on a Bruker AV-300, equipped with a 4 mm MAS probe. The signal resonances are collected into six main spectral regions related to the classes of carbon functional moieties. The combination of specific spectral regions provides the calculation of dimensionless structural descriptors of molecular features of HS, namely Hydrophobic (HB) and Aromatic (Ar) index, Alkyl (A/OA) and Lignin (LR) ratio [10, 18].

### Thermochemolysis Gas_Chromatography Mass_Spectrometry

The molecular components of compost extracts have been detached from the organic network by off-line pyrolysis (THM) and subsequently detected by Gas Chromatography Mass Spectrometry (GC_MS) analysis [10, 18]. About 100 mg of each humic substance was placed in a quartz boat, moistened with 0.5 ml of tetramethylammonium hydroxide (25% in methanol) solution, and introduced into a Pyrex tubular reactor (50 cm×3.5 cm) heated at 400 °C for 30 min in a furnace. The components released by thermochemolysis were transferred by a helium flow (20 mL/min) into two successive chloroform (50 mL) traps. The chloroform solutions were combined and concentrated by roto-evaporation under reduced pressure. The residue was hence re-dissolved in 1 ml of chloroform and transferred to a glass vial for GC–MS analysis (PerkinElmer, Auto System XL) using an RTX-5MS WCOT capillary column (Restek, 30 m ∅ 0.25 mm; film thickness = 0.25 μm) coupled to a PE Turbomass-Gold quadrupole mass spectrometer. The dissolved samples introduced in the GC at an inlet temperature of 250 °C, underwent chromatographic separation with a ramp program (T1 = 60 °C held for 1 min, ramp 1 = 7 °C min^–1^ up to T2 = 100 °C, ramp 2 = 4 °C min^–1^ up to T3 = 300 °C held for 10 min). Helium was used as a carrier gas at 1.00 mL min^–1^ with a split ratio of 1:30. Mass spectra were obtained in EI mode (70 eV), scanning in the range of m/z 45–650 for a cycle time of 0.2 s. The pyrolytic products were identified and classified according to mass spectra and the NIST database.

### Antioxidant assay

Due to the heterogeneity and complexity of humic products, antioxidant activity is better evaluated by applying two complementary spectrophotometric methods [11]. Here, 2,20 -azino-bis (3-ethylbenzothiazoline-6-sulphonic acid (ABTS) and Ferric reducing ability of plasma (FRAP) were employed to evaluate the antioxidant features of HS-OL and HS-CYN. Briefly, the ABTS assay has been carried out as described in Verrillo et al., 2021 [10]. Conversely, FRAP determination was performed using a FRAP reagent prepared before each measurement by mixing acetate buffer (300 mM), TPTZ (10 mM), and FeCl_3_ (20 mM in Milli Q Water) and incubated at 37 °C for 10 min. For the analysis, 2 mL of FRAP solution was mixed with sample, measuring the absorbance at 593 nm. A calibration curve with six Trolox standards was employed [12].

### Antimicrobial assay

Assessment of antimicrobial capacities of humic product has been performed by the diffusion disk test (National Committee for Clinical Laboratory Standards (NCCLS) method). Microbial cells include *Helicobacter pylori* (Hp), *Salmonella typhi ATCC14028* (S.T.), *Staphylococcus epidermis* (S.E.) and *Listeria monocitogenes* ATCC19115 (L.M.). The inoculum of each microbial cell was carried out in nutrient agar with sterile saline buffer up to 10^8^ CFU/mL (0.5 McFarland). Then, 300 μL of each microorganism was placed on Mueller–Hinton agar. Finally, six disks (6.0 mm diameter) were treated with 20 µg of each humic substance, placed on the agar, and incubated at 37 °C for 24 h. Ampicillin (AMP), Clavulanic Acid and Bovine serum albumin (BSA) were employed as negative control and positive reference, respectively.

### Cell culture

CR-CSphC #2, CR-CSphC #9 and B-CSphC #21 were obtained from surgical tumor tissue. Cell purification and propagation were assessed as previously described [23, 24], and DNA profiles were matched with their relative patient tumor tissues. B-CSphC #21 cells were isolated from a Her2-enriched tumor specimen, which was serially transplanted in immunocompromised mice, and displayed an enrichment in the stem cell compartment (CD44^high^/CD24^low^). B-CSphC #21 were cultured in serum-free stem cell medium (SCM) supplemented as reported in previous work [23].

RKO, 8505, MDA-MB231 and HUVEC cells were obtained from ATCC (Manassas, VA, USA) and cultured according to the manufacturer’s instructions. h-TERT-immortalized IMEC, mammary epithelial cells, were provided by Prof. Alessio Zippo and cultured according to the manufacturer’s instructions. Mycoplasma presence was assessed with the MycoAlertTM Plus Mycoplasma Detection Kit (Lonza, Houston, TX, USA). Routinely, cell authentication was performed using a short tandem repeat DNA profiling kit (GlobalFiler™ PCR kit, Applied Biosystem) following the manufacturer’s instructions and analyzed by ABI PRISM 3130 (Applied Biosystem).

### In vitro treatment of primary and established cell lines

CR-CSphCs and RKO were treated with HS-OL and HS-CYN 500 μg/ml, FOLFOX [5-Fluorouracil 1,25 μM (Selleckchem), Oxaliplatin 1,25 μM (Sigma-Aldrich) and Leucovorin 1,25 μM (Selleckchem)], FOLFIRI [5-Fluorouracil 1,25 μM (Selleckchem), Irinotecan 1,25 μM (Selleckchem) and Leucovorin 1,25 μM (Selleckchem)] alone or in combination. *NRAS/TP53* TPCs were treated with HS-OL and HS-CYN 125 μg/ml and doxorubicin 50 nM (Selleckchem). 8505c were treated with HS-OL and HS-CYN 500 μg/ml and doxorubicin 200 nM (Selleckchem). B-CSphC #21 and MDA-MB-231 were treated with HS-OL and HS-CYN 350 μg/ml and doxorubicin 200 or 50 nM, respectively. All the compounds were replenished in culture media every 48 h.

### Cell viability

To determine HSs’ IC50, 2 × 10^3^ cells were seeded in 96-well plates and treated with different concentrations (0–16–31–62–125–250 and 500 µg/ml) up to 48 h. Cell viability assays were assessed by CellTiter-Glo^®^ Luminescent Cell Viability Assay kit (Promega Madison, WI, USA) according to the manufacturer’s instructions and analyzed by Infinite® F500 (Tecan).

8505c cells, pre-treated with HS-OL or HS-CYN for 48 h, were irradiated with 5 Gy at room temperature. Following irradiation, cells were seeded in complete culture medium in 6-well plates (20,000 cells/well) with or without the addition of HS-OL or HS-CYN. After 21 days, the cells were fixed with 4% paraformaldehyde and stained with 0.1% crystal violet-methanol solution (Sigma-Aldrich). Cell proliferation was quantified using ImageJ software.

### Flow cytometry analysis

CR-CSphC #2, RKO, *NRAS/TP53* TPCs, 8505c, MDA-MB 231 and B-CSphC #21 cells treated with HSs were washed with PBS and centrifuged at 1300 rpm for 5 min. The cell pellet was resuspended in 1 mL of Nicoletti Buffer, which consisted of 0.1% sodium citrate, 0.01% Triton X-100, 50 μg/mL propidium iodide, and 10 μg/mL RNase solution. The resuspended cells were incubated in the dark at 4 °C for 16 h. Apoptotic cells were detected by using the CaspGlow Fluorescein, Active Caspase 3 Staining kit (Catalog number: K193-100, Lot: 7A20K01930, Biovision, Milpitas, CA, USA) according to the manufacturer’s protocol.

FACS Lyric flow cytometer (BD Biosciences, Franklin Lakes, NY, USA) was used for cell detection, and FlowJo ^TM10^ software was used for the analysis.

### Western blot

CR-CSphC #9, RKO, *NRAS/TP53* TPCs, 8505c, MDA-MB-231 and B-CSphC #21 cells were harvested by scraping in ice-cold PBS and resuspended in ice-cold F buffer (Tris-HCL 10 mM, NaCl 50 mM, sodium pyruvate 30 mM, NaF 50 nM, ZnCl2 5 μM, triton 1, sodium orthovanadate 0.1 nM, sodium butyrate 10 mM and PMSF 1 mM) supplemented with protease and phosphatase inhibitors (Sigma-Aldrich). Whole-cell lysates were loaded in sodium dodecyl sulfate-polyacrylamide-gel electrophoresis gels and blotted on nitrocellulose membranes. Membranes were blocked with a 5% nonfat dry milk and 0.1% Tween 20 PBS solution for 1 h at room temperature and then incubated with specific antibodies against Phospho-Histone H2A.X (γ-H2AX, Ser139, 20E3, Catalog number: #9718, Lot:21, Rabbit IgG, Cell Signaling Technology) and beta-Actin (8H10D10) (Catalog number: #3700, Lot:21, Mouse IgG2b, Cell Signaling Technology). Primary antibodies were revealed using anti-Mouse (Goat IgG, H + L, Catalog number: 32430, Lot: UJ290644, Invitrogen) or anti-Rabbit (Goat IgG, H + L, Catalog number: 32460, Lot: ZG401527, Invitrogen) HRP-conjugated and detected by Amersham imager 600 (GE Healthcare). Protein levels were normalized with β-actin and calculated by densitometric analysis using ImageJ software.

### RNA isolation and gene expression analysis

Total RNA was obtained from CR-CSphC #2, 8505c and B-CSphC #21 cells using TRIzol (Thermo Fisher Scientific, Waltham, MA, USA) treated with HSs for 48 h. RNA concentration was determined with the NanoDrop™ 1000 Spectrophotometer (Thermo Fisher Scientific). For gene expression analysis, 1 µg of RNA was retrotranscribed using a PrimePCR custom panel (Bio-Rad, Hercules, CA, USA) following the manufacturer’s instructions. Gene expression was analyzed using a PrimePCR-designed panel (Bio-Rad) targeting genes involved in the DNA damage signaling pathway. The panel included 89 genes involved in different mechanisms of DNA damage repair, including base excision repair, nucleotide excision repair, mismatch repair, and double-strand break repair pathways. Gene expression levels were normalized to the two housekeeping genes *GAPDH* and *HPRT1*. For Real-time PCR, RNA was retrotranscribed using the High-Capacity cDNA Reverse Transcription Kit (Applied Biosystems), and qRT-PCR was performed using specific primers. Quantitative Real-time PCR analysis was performed in a SYBR Green Master Mix (Qiagen, 1054586) using primers for Hs-*RAD50* (FW: GGAAGAGCAGTTGTCCAGTTACG; REV: GAGTAAACTGCTGCTCCAG), Hs-*GAPDH* (FW: GCTTCGCTCTCTGCCTCCTCC; REV: ACCACCCTGTTGCTGTAGCCAA), Hs-*CDK7 (*FW: *GCACACCAACTGAGGAACAGTG; REV: AAGTCGTCTCCTGCTGCACTGA, Hs-DDB2 (*FW*: CCAGTTTTACGCCTCCTCAATGG; REV: GGCTACTAGCAGACACATCCAG)*, Hs*-ERC2 (*FW*: CAAGACGAAGGCTCTCCAGACT; REV: CAGTGTTCAGCACACCATTGGC)*, Hs*-MBD4* (FW*: CTGAGGTAGCAAGAACCGCAGA;* REV: GGATACTTCCACTGCTTTGTCAG), Hs- *PTGES (*FW: *GAGGATGCCCTGAGACACGGA;* *REV: CCAGAAAGGAGTAGACGAAGCC)*, Hs*-PRKDC (*FW*: GCGCCATATCTGTCATCTGCTG; REV: TTATAGCGGCGCTTCAGGTCGA)*, Hs*-CIB1* (FW: *ACACAGCCACGCCAGACATCAA; REV: GCTGCTTCATCTCAGACGCACT)*, Hs*-RNF8 (*FW*: GGAGAAAAGGACCTGAAGCAACA; REV: GCTTCAAAGTCCTTCTTGCTGCG)*, Hs*-MPG (*FW*: GGTTGGAGTTCTTCGACCAGCC; REV: GTATGCCTCGGTCTCCACGAT)*.

### Drug combination study

Drug combination studies have been assessed by using the Chou–Talalay method, which is based on the median effect and the combination index (CI) equations to determine the quantification of drug interactions. The CI, computed in CompuSyn using the Chou–Talalay method, was calculated on CR-CSphC #2, 8505c and B-CSphC #21 cells treated as indicated for 48 h.

CI < 1 represented synergism (slight, moderate, strong, very strong); otherwise, it indicated additivity (CI=1) or antagonism (CI > 1) between two drugs.

### Immunofluorescence analysis

Immunofluorescence analysis was performed on CR-CSphC #9, RKO, *NRAS/TP53* TPCs, 8505c, MDA-MB-231 and B-CSphC #21 cells treated with HS-OL and HS-CYN for 48 h. Staining was carried out with Cleaved Caspase-3 (Asp175) Antibody (Catalog number: #9661, Lot: 47, Rabbit, Cell Signaling Technology) revealed by Alexa Fluor 488-conjugated secondary antibody; Phospho-Histone H2A.X (γ-H2AX, Ser139, 20E3, Catalog number: #9718, Lot:21, Rabbit IgG, Cell Signaling Technology) revealed by Alexa Fluor 555-conjugated secondary antibody (Catalog number: R6394, Lot: I702286, IgG, H + L, Invitrogen). Activated-3 Caspase and Y-H2AX positive cells were counted with ImageJ Cell Counting software. Nuclei were counterstained using Hoechst (Thermofisher).

### Statistical analysis

All experiments were conducted at least twice, and the results are presented as the mean ± standard deviation. Statistical significance was estimated by an unpaired t-test. Results were referred to as statistically significant, as *p* < 0.05. ∗ indicates *p* < 0.05, ∗∗ indicate *p* < 0.01, ∗∗∗ indicate *p* < 0.001, and ∗∗∗∗ indicate *p* < 0.0001.

## Supplementary information

Supplementary Figure 1.

Supplementary Figure 2.

Supplementary Figure 3.

Supplementary Figure 4.

Supplementary Figure 5.

Original data

## Data Availability

All relevant raw data will be available on request from the corresponding author.
