# Supplementary figures and images for: Humic substances enhance the anti-cancer efficacy of standard therapies
Source: Cell Death Discov. 2026 Mar 31;12:207. doi: 10.1038/s41420-026-03083-1 (PMC13158296; doi:10.1038/s41420-026-03083-1)

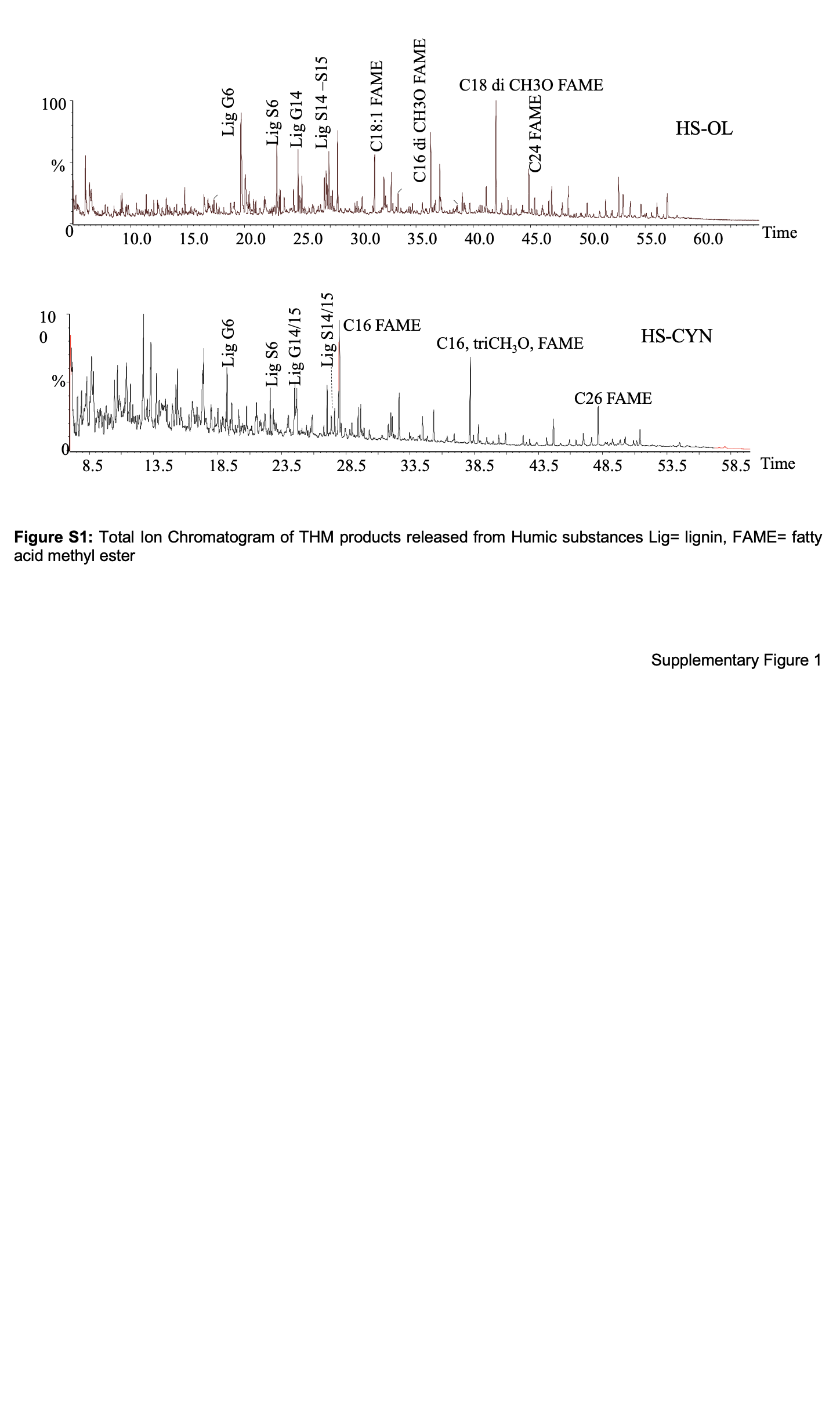

Supplement: Supplementary file 1 — Supplementary Figure 1. [file 41420_2026_3083_MOESM1_ESM.png]

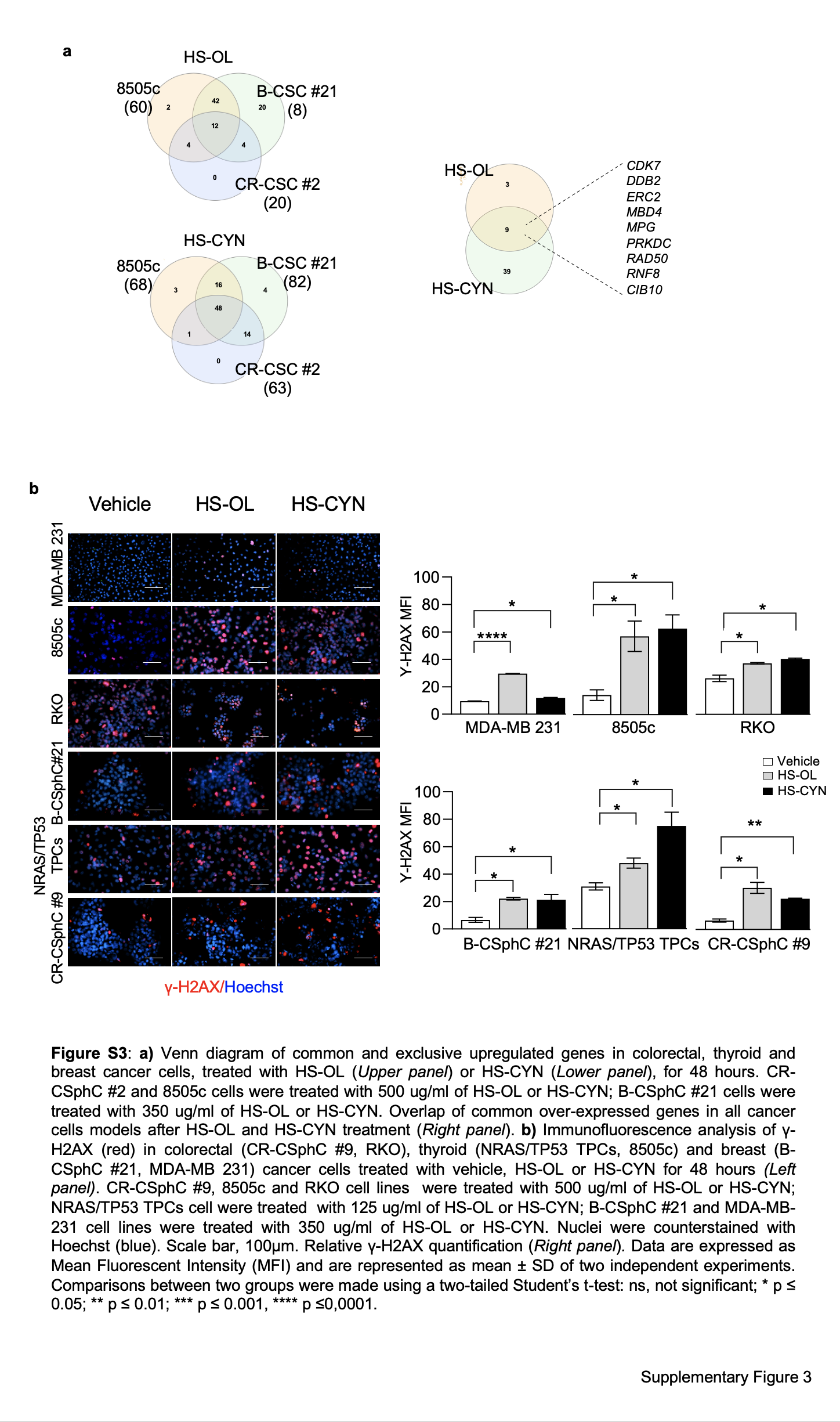

Supplement: Supplementary file 3 — Supplementary Figure 3. [file 41420_2026_3083_MOESM3_ESM.png]

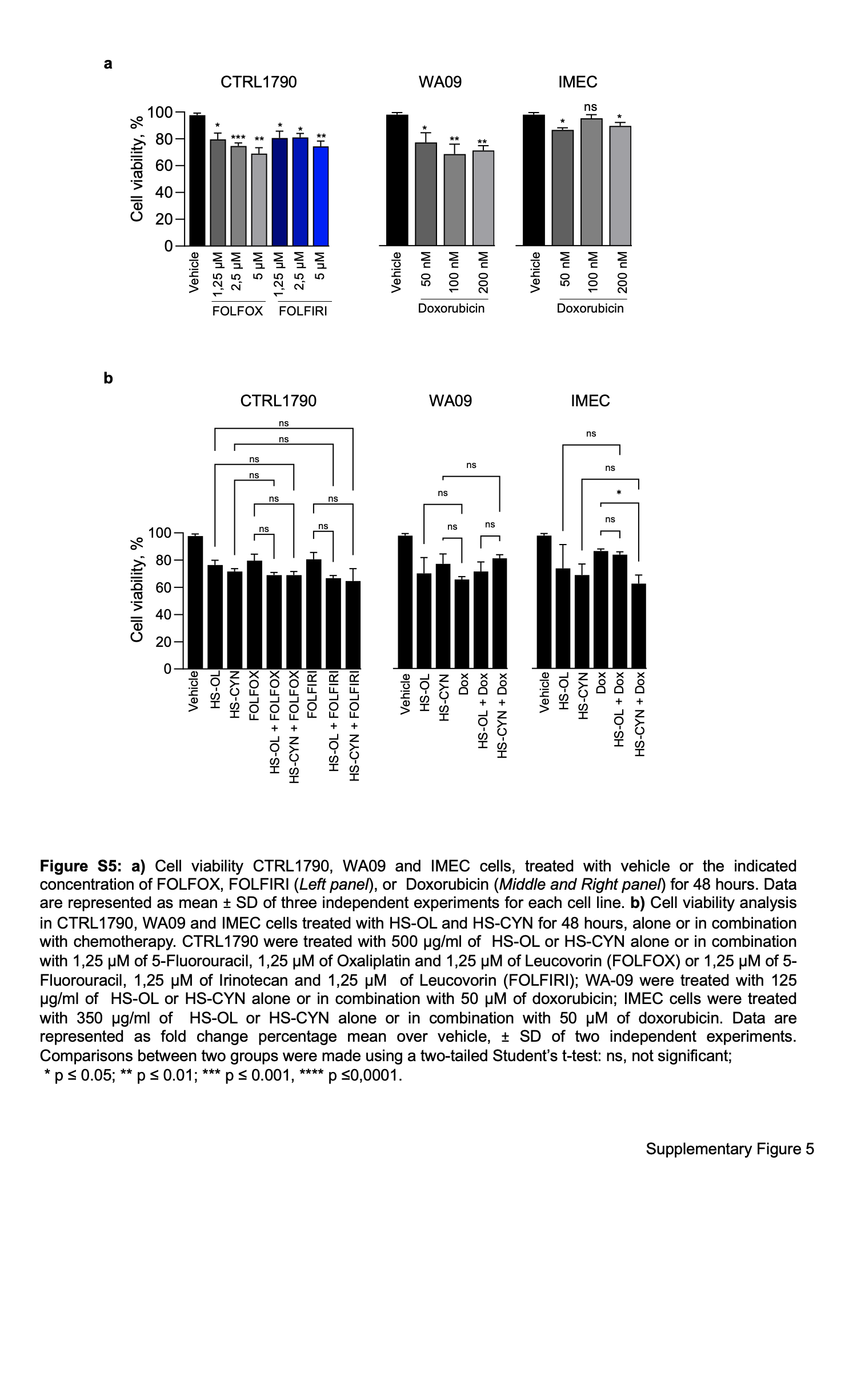

Supplement: Supplementary file 5 — Supplementary Figure 5. [file 41420_2026_3083_MOESM5_ESM.png]
