# Supplementary material for: Humic substances enhance the anti-cancer efficacy of standard therapies
Source: Cell Death Discov. 2026 Mar 31;12:207. doi: 10.1038/s41420-026-03083-1 (PMC13158296; doi:10.1038/s41420-026-03083-1)
Supplement: Supplementary file 2 — Supplementary Figure 2. [file 41420_2026_3083_MOESM2_ESM.pdf]

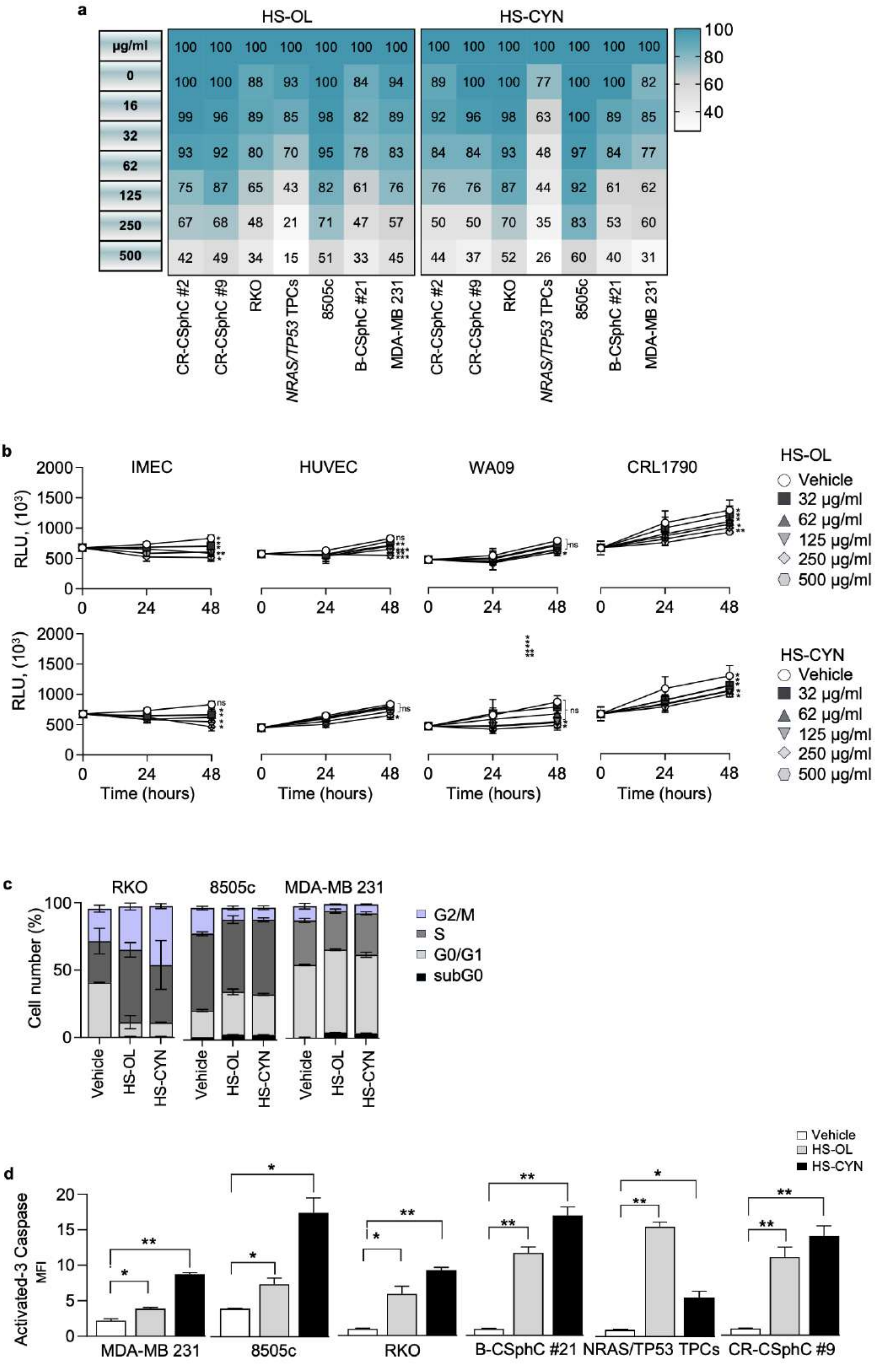

Supplementary Figure 2

Figure S2: a) Dose response assay in colorectal (CR-CSphC #2, CR-CSphC #9, RKO), thyroid (NRAS/TP53 TPCs, 8505c), and breast (B-CSphC #21, MDA-MB 231) cancer cells, treated with HS-OL (*Left panel*) and HS-CYN (*Right panel*) at the indicated concetrations (16 – 32 – 62- 125 – 250 and 500 µg/ml) for 48 hours. **b)** Cell viability assay of IMEC, HUVEC, WA09 and CRL1790 cells treated with HS-OL (*Upper panel*) and HS-CYN (*Bottom panel*) at the indicated concentrations (32 – 62- 125 – 250 and 500 µg/ml) up to 48 hours. Data are represented as mean ± SD of three different experiments. **c)** Cell cycle analysis in RKO, 8505c, MDA-MB 231 established cell lines treated with vehicle, HS-OL or HS-CYN for 48 hours. RKO and 8505c were treated with HSs 500mg/ml, MDA-MB 231 were treated with HSs 350mg/ml. The percentages of cells in sub-G0, G1, S, and G2 phases are shown as mean ± SD from two independent experiments. **d)** Quantification of immunofluorescence staining of activated-3 caspase of colorectal (CR-CSphC #9, RKO), thyroid (NRAS/TP53 TPCs, 8505c), and breast cancer cells (B-CSphC #21, MDA-MB-231), treated with vehicle, HS-OL or HS-CYN for 48 hours. CR-CSphC #9, 8505c and RKO cell lines were treated with 500 ug/ml of HS-OL or HS-CYN; NRAS/TP53 TPCs cell were treated with 125 ug/ml of HS-OL or HS-CYN; B-CSphC #21 and MDA-MB-231 cell lines were treated with 350 ug/ml of HS-OL or HS-CYN. Data are expressed as Mean Fluorescent Intensity (MFI) and are represented as mean ± SD of two independent experiments..

Comparisons between two groups were made using a two-tailed Student's t-test: ns, not significant; * $p \leq 0.05$; ** $p \leq 0.01$; *** $p \leq 0.001$.
