# Supplementary material for: Humic substances enhance the anti-cancer efficacy of standard therapies
Source: Cell Death Discov. 2026 Mar 31;12:207. doi: 10.1038/s41420-026-03083-1 (PMC13158296; doi:10.1038/s41420-026-03083-1)
Supplement: Supplementary file 4 — Supplementary Figure 4. [file 41420_2026_3083_MOESM4_ESM.pdf]

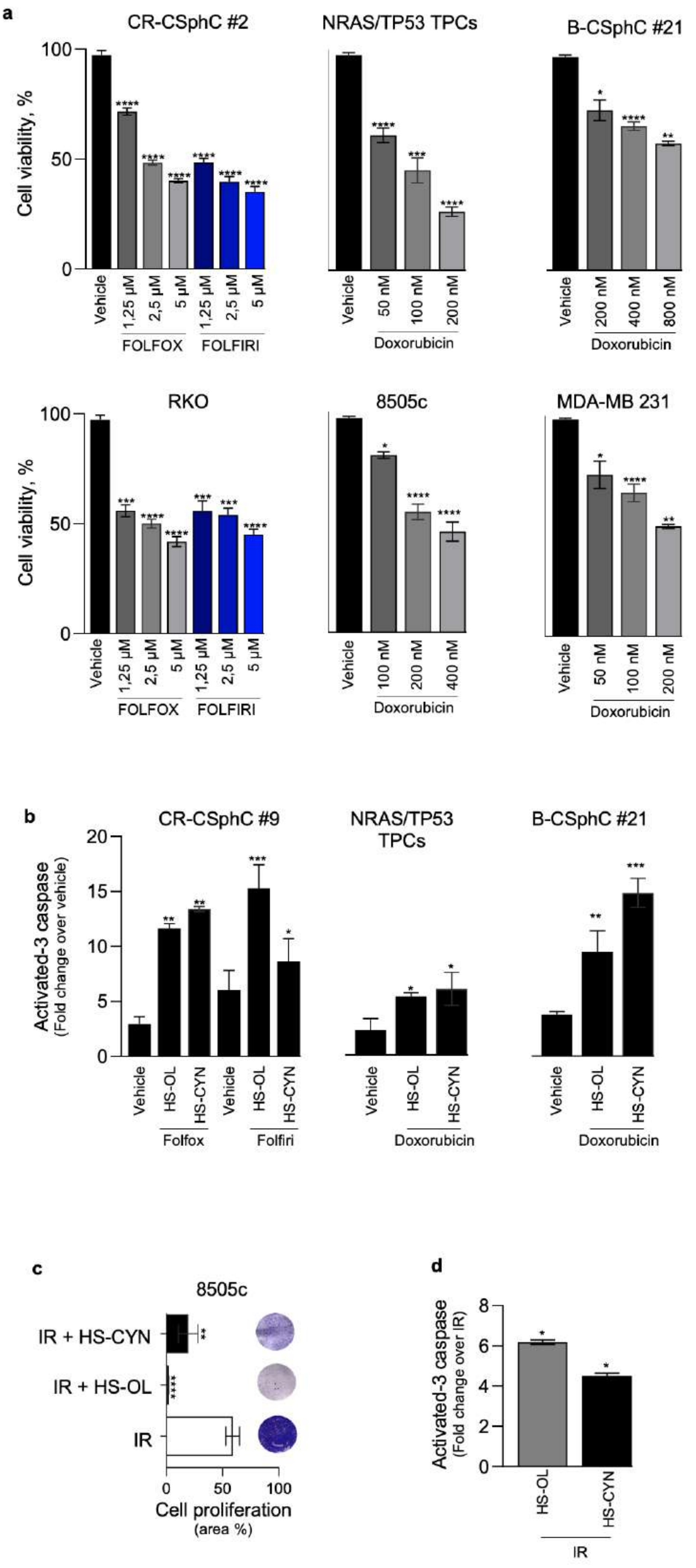

a
CR-CSphC #2
NRAS/TP53 TPCs
B-CSphC #21
Cell viability, %
100
50
0
Vehicle
1,25 µM
2,5 µM
5 µM
FOLFOX
FOLFIRI
50 nM
100 nM
200 nM
Doxorubicin
400 nM
800 nM
RKO
8505c
MDA-MB 231
b
CR-CSphC #9
NRAS/TP53 TPCs
B-CSphC #21
Activated-3 caspase
(Fold change over vehicle)
20
15
10
5
0
HS-OL
HS-CYN
Folfox
Folfiri
Doxorubicin
c
8505c
IR + HS-CYN
IR + HS-OL
IR
Cell proliferation
(area %)
d
Activated-3 caspase
(Fold change over IR)
8
6
4
2

Figure S4: a) Cell viability of colorectal (CR-CSphC#2, RKO) thyroid (NRAS/TP53 TPCs, 8505c) and breast (BCSphC#21, MDA-MB 231) cancer cells, treated with vehicle or the indicated concentration of FOLFOX, FOLFIRI, or Doxorubicin for 48 hours. Data are represented as mean ± SD of three independent experiments for each cell line. **b)** Activated-3 caspase flow cytometry analysis in CR-CSphC #9, NRAS/TP53 TPCs and B-CSphC #21 cell lines treated as indicated for 48 hours. CR-CSphC #9 were treated with 500 µg/ml of HS-OL or HS-CYN alone or in combination with 1,25 µM of 5-Fluorouracil, 1,25 µM of Oxaliplatin and 1,25 µM of Leucovorin (FOLFOX) or 1,25 µM of 5-Fluorouracil, 1,25 µM of Irinotecan and 1,25 µM of Leucovorin (FOLFIRI); NRAS/TP53 TPCs were treated with 125 µg/ml of HS-OL or HS-CYN alone or in combination with 50 µM of doxorubicin; B-CSphC #21 cells were treated with 350 µg/ml of HS-OL or HS-CYN alone or in combination with 50 µM of doxorubicin. Data are represented as fold change percentage mean over vehicle, ± SD of three independent experiments for each cell line. **c)** Proliferation assay of irradiated (5Gy) 8505c cancer cells in presence of HS-OL or HS-CYN (500 µg/ml). Data are represented as mean of percentage area ± SD of two independent experiments. **d)** Activated-3 caspase flow cytometry analysis in 8505c treated as in c. Data are represented as fold change percentage mean over irradiated cells, ± SD of two independent experiments. Comparisons between two groups were made using a two-tailed Student's t-test: ns, not significant; * $p \leq 0.05$; ** $p \leq 0.01$; *** $p \leq 0.001$, **** $p \leq 0{,}0001$.

Supplementary Figure 4
