# Supplementary material for: Humic substances enhance the anti-cancer efficacy of standard therapies
Source: Cell Death Discov. 2026 Mar 31;12:207. doi: 10.1038/s41420-026-03083-1 (PMC13158296; doi:10.1038/s41420-026-03083-1)

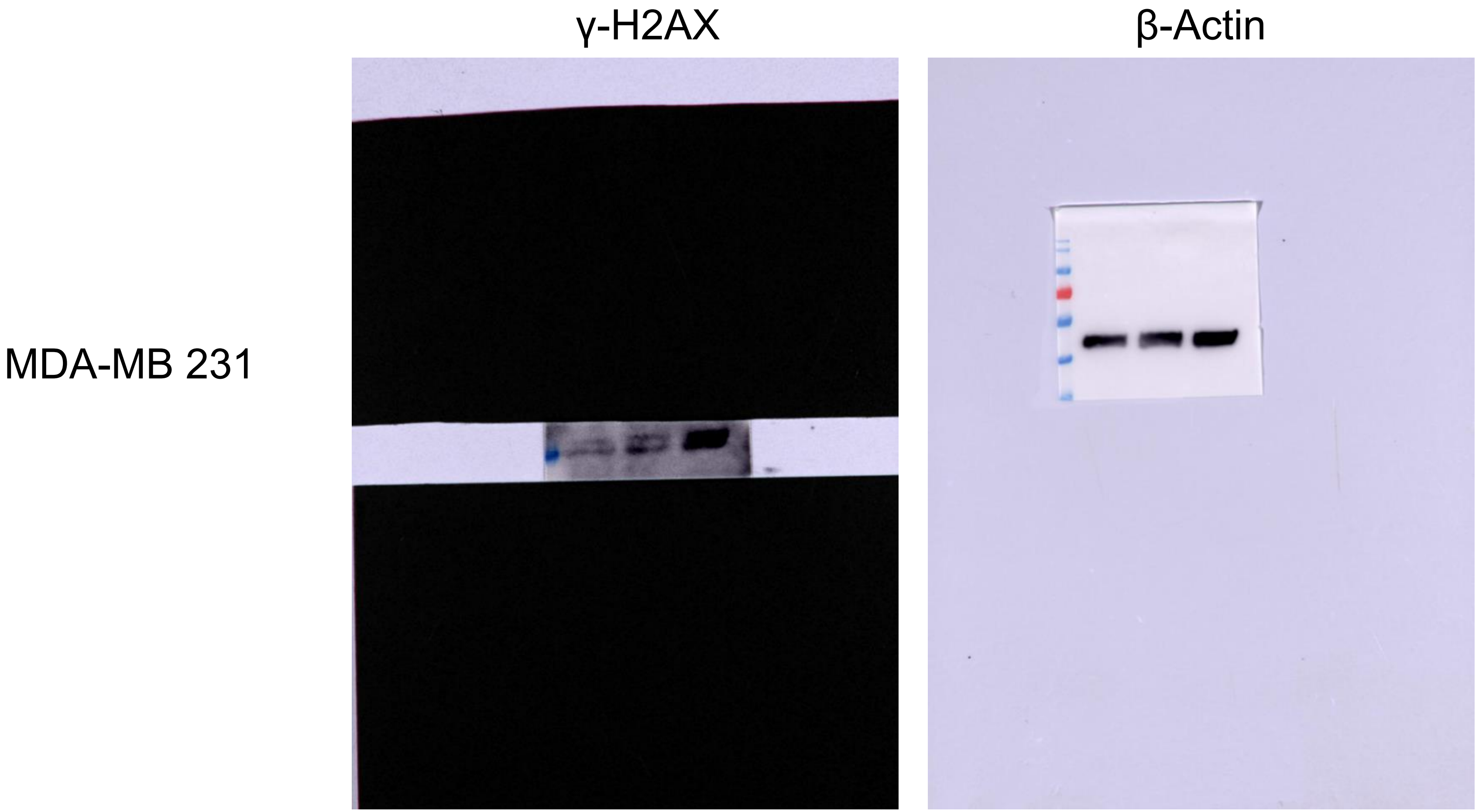
γ-H2AX
β-Actin
MDA-MB 231

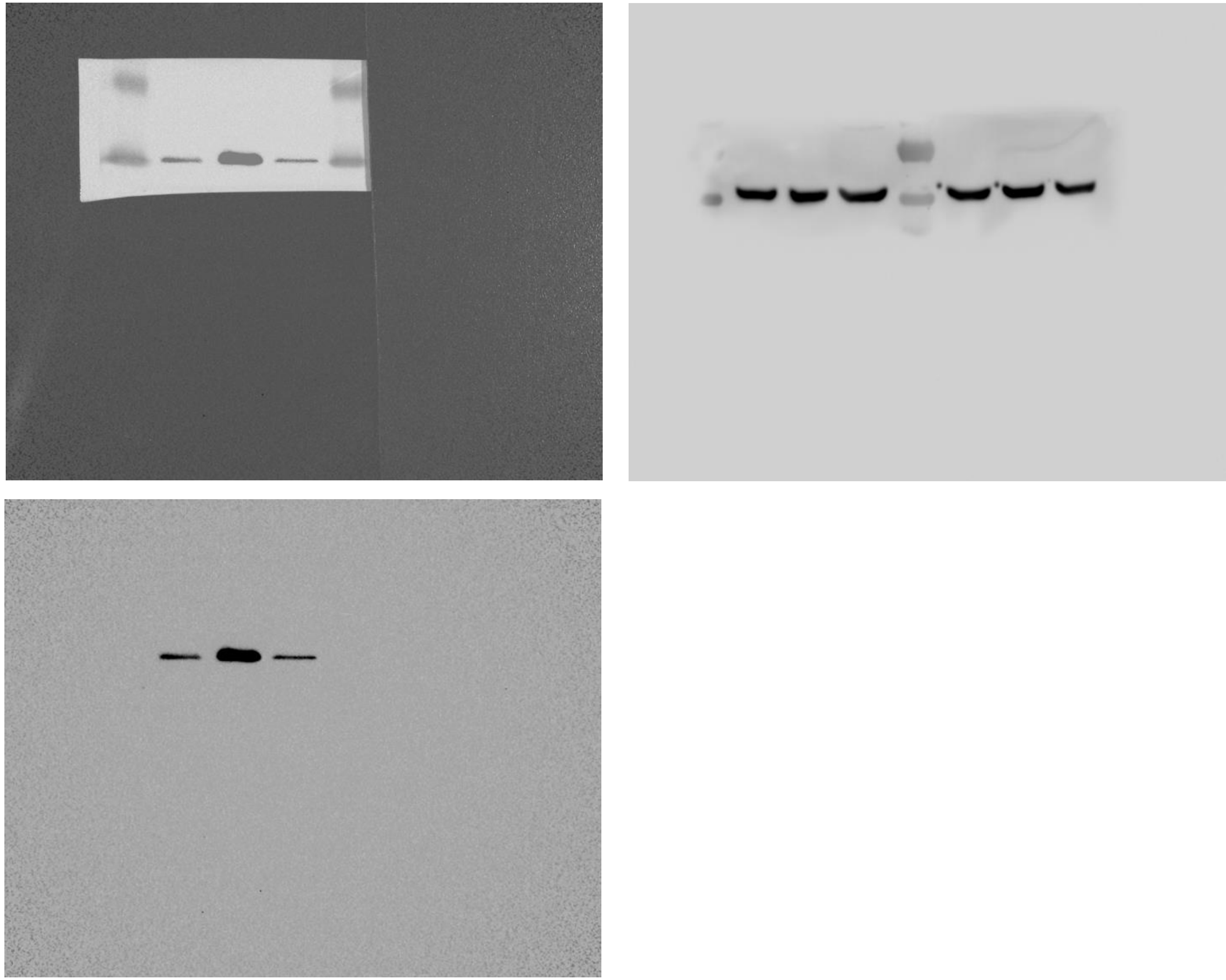
γ-H2AX
β-Actin
NRAS/TP53
TPCs

γ-H2AX

β-Actin

8505c

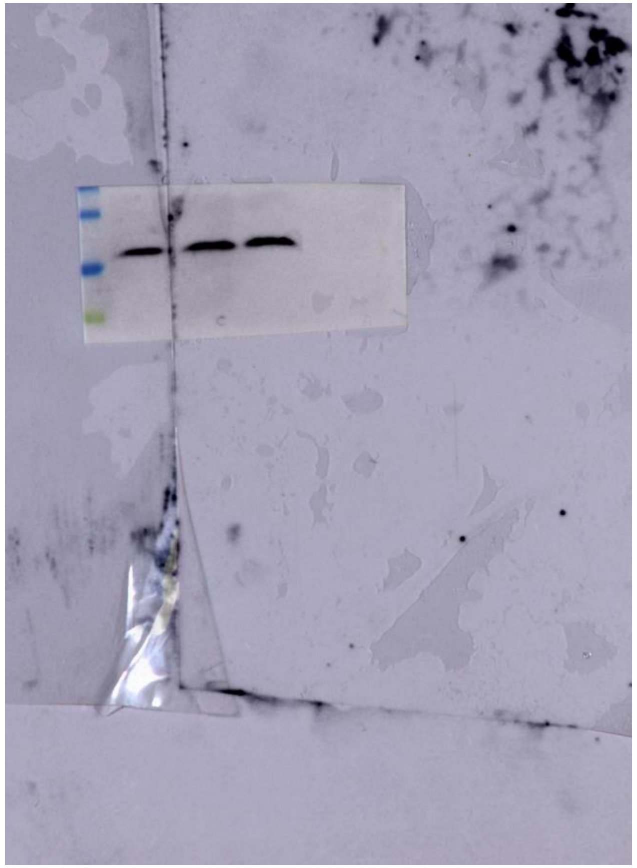

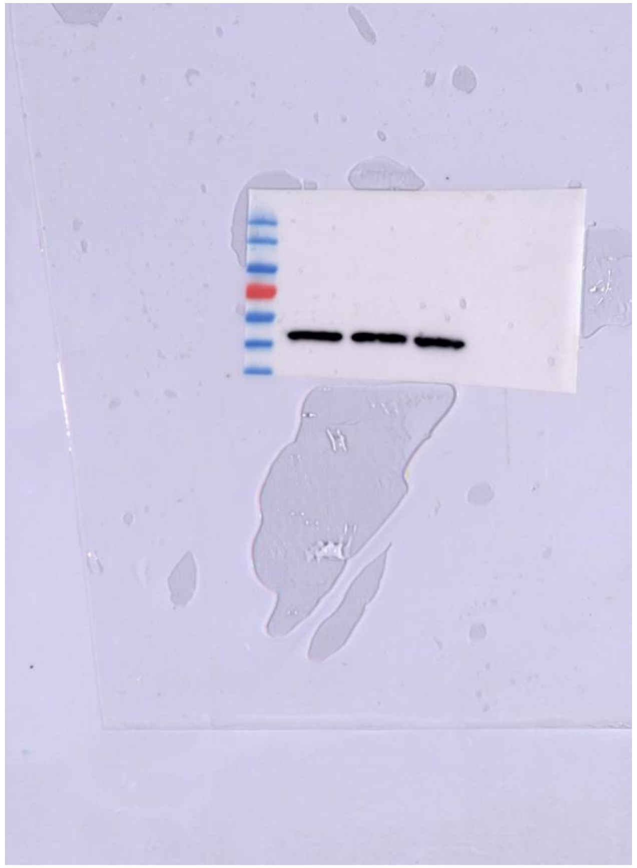

RKO

γ-H2AX

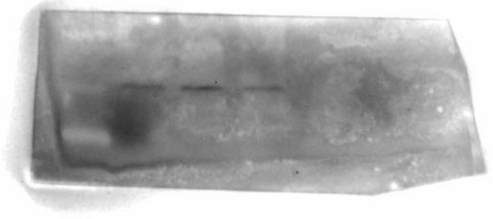

β-Actin

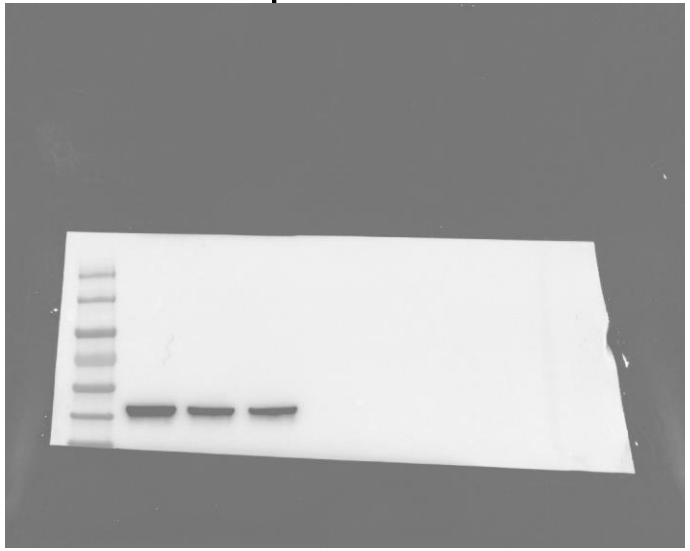

γ-H2AX

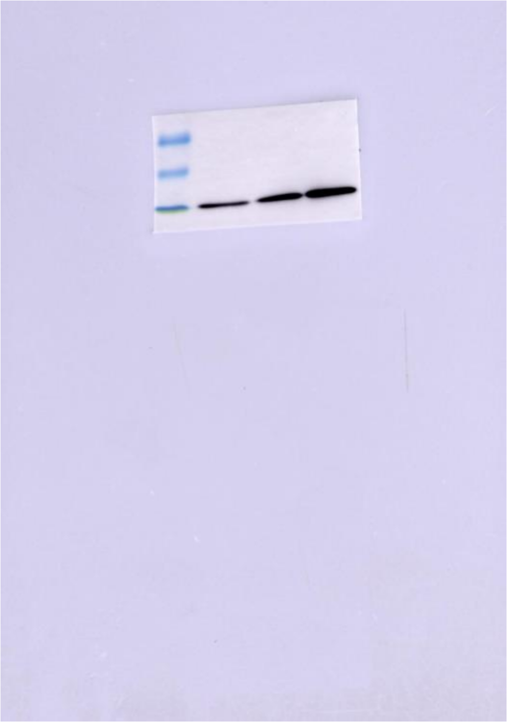

β-Actin

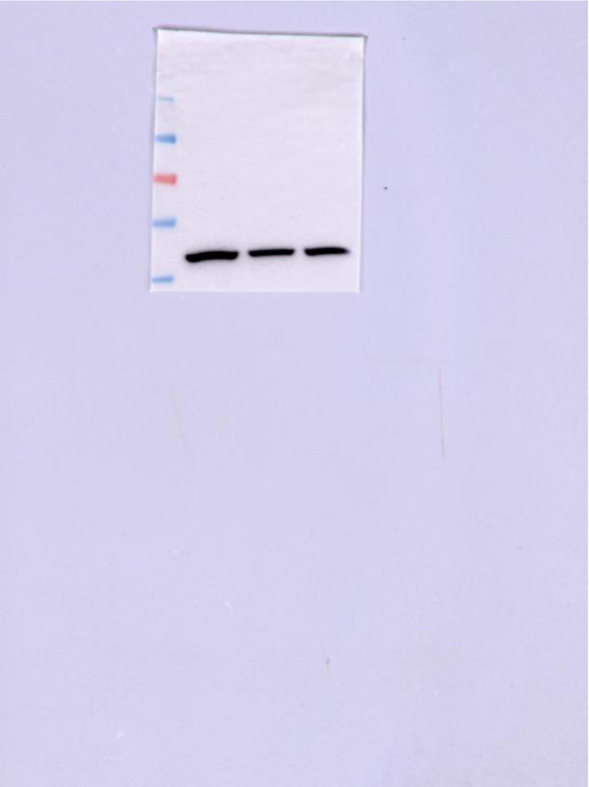

CR-CSphC #9

γ-H2AX

β-Actin

B-CSphC #21

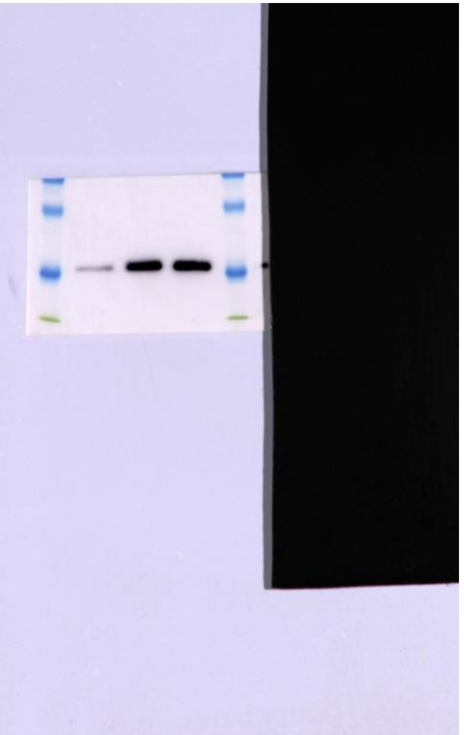

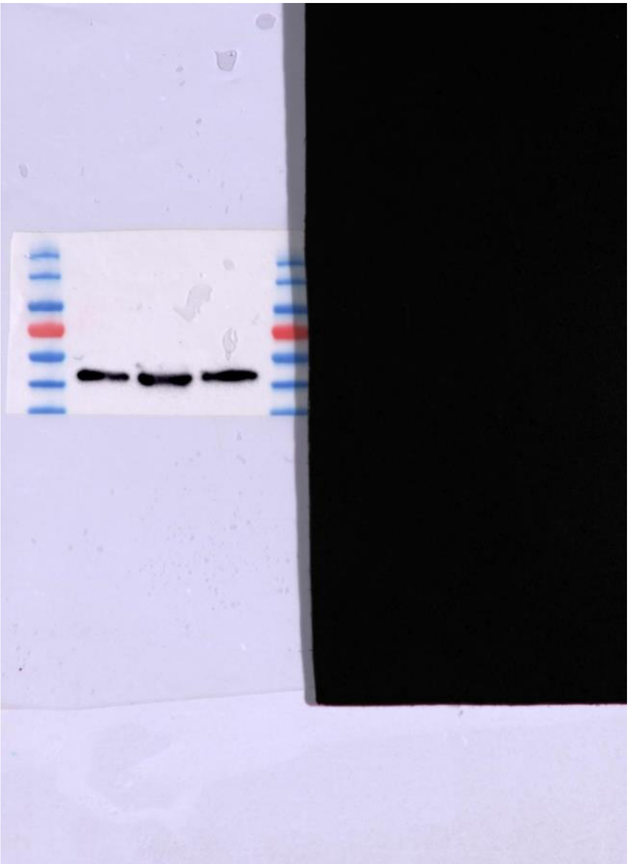

Supplement: Supplementary file 6 — Original data [file 41420_2026_3083_MOESM6_ESM.pdf]
